# Supplementary material for: An adverse tumor-protective effect of IDO1 inhibition
Source: Cell Rep Med. 2023 Feb 21;4(2):100941. doi: 10.1016/j.xcrm.2023.100941 (PMC9975322; doi:10.1016/j.xcrm.2023.100941)
Supplement: Document S1. Figures S1–S3 [file mmc1.pdf]

Cell Reports Medicine, Volume *4*

Supplemental information

An adverse tumor-protective effect of IDO1 inhibition

Juliana C.N. Kenski, Xinyao Huang, David W. Vredevoogd, Beaunelle de Bruijn, Joleen J.H. Traets, Sofía Ibáñez-Molero, Sebastiaan M. Schieven, Alex van Vliet, Oscar Krijgsman, Thomas Kuilman, Joanna Pozniak, Fabricio Loayza-Puch, Alexandra M. Terry, Judith Müller, Meike E.W. Logtenberg, Marjolein de Bruijn, Pierre Levy, Pierre-René Körner, Colin R. Goding, Ton N. Schumacher, Jean-Christophe Marine, Reuven Agami, and Daniel S. Peeper

Supplemental file.

Figure S1

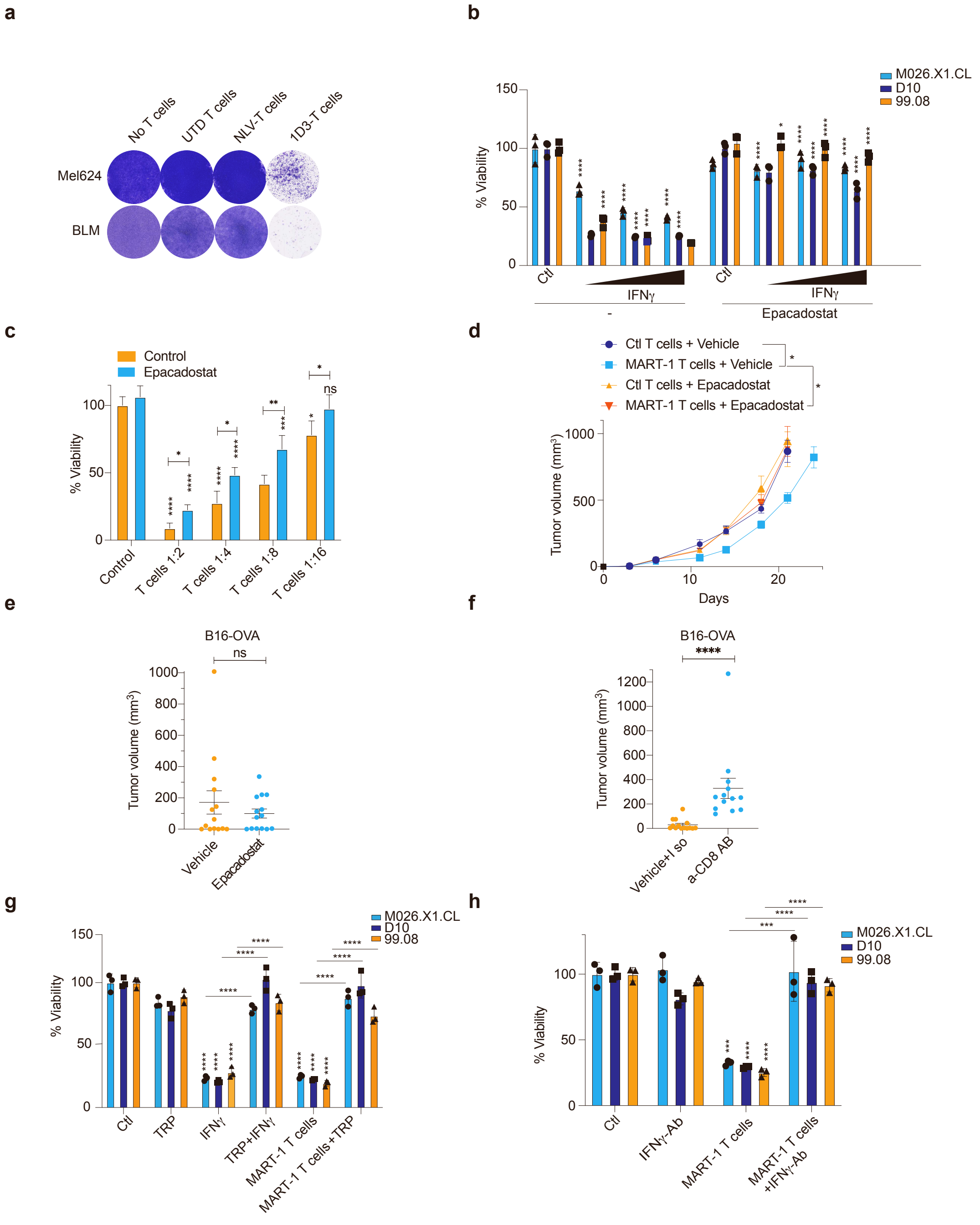

Figure S1 (related to Fig. 1). Tryptophan restoration by IDO1 inhibition protects tumor cells to T cell-mediated killing. a, Mel624 and BLM melanoma cell lines were exposed to 1D3-specific (MART-1) T cells for 24 hrs. Cells were stained with crystal violet after 6 days. Untransduced T cells and T cells containing the CMV-specific TCR (NLV) were used as controls to determine TCR-specific killing. **b**, Quantification of Fig. 1e. Statistical testing of the IFNγ only group was performed against its control, whereas in the epacadostat-treated groups it was compared to the corresponding IFNγ dose**. c,** A375-MelanA cells were co-cultured with MART-1 T cells at indicated effector to target ratios in the presence or absence of epacadostat (2uM). Cells were fixed and stained with crystal violet after 6 days. Graph shows quantification of the remaining viable melanoma cells. Statistical testing was done to compare both the effect of T cells against control (untreated) and T cells versus T cell+Epacadostat in each respective ratio. **d,** Growth curves of in vivo experiment shown in Fig. **1f** . **e,** B16-F10 melanoma cells expressing the model antigen ovalbumin (OVA) were injected into immunocompetent Black6 mice (n=14) and treated daily with either epacadostat (100mg/kg) or vehicle control by oral gavage. Tumors were measured three times a week and individual tumor sizes for the last time point when all the mice were alive. Statistical significance tested by Mann-Whitney. **f,** CD8-depleting antibody and isotype control were injected into immunocompetent Black6 mice until $CD8^+$ T cells were depleted (confirmed in the blood of treated mice by flow cytometry). B16-F10 melanoma cells expressing the model antigen ovalbumin (OVA) were injected after depletion was confirmed and tumors were measured three times a week and average tumor size +/- SEM are plotted in the graph. Graph shows individual tumor sizes for the last time point when all the mice from both groups were alive. Statistical significance tested by Mann-Whitney. In e, f, the experiment was done in parallel, but they have different time points due to distinct endpoints of each experiment. **g**, Quantification of Fig. 1h. Statistical testing of IFNγ and MART-1 T cell only groups was performed against their own controls. **h**, Quantification of Fig. 1j. Bars represent +/- SD for *in vitro* and SEM for *in vivo*. Statistical testing was performed on the three technical replicates by one-way Anova with Tukey's *Post-hoc* test. *= $p \leq 0.05$; **= $p \leq 0.01$; ***= $p \leq 0.001$; ****= $p \leq 0.0001$. One-way Anova with Šidák's Post-Hoc for **c, d**. *In vitro* experiments (except 1a) were performed in two biological replicates with three technical replicates each.

Figure S2

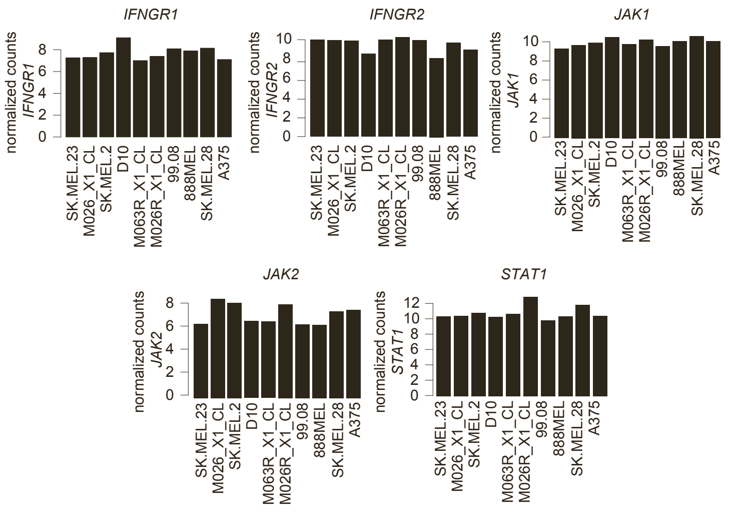

Figure S2 (related to Fig. 3). Expression of IFNγ pathway components by RNA-Sequencing. *IFNγR1, IFNγR2, JAK1, JAK2* and *STAT1* RNA levels were determined by RNA sequencing, which was performed in 2 independent biological replicates.

Figure S3

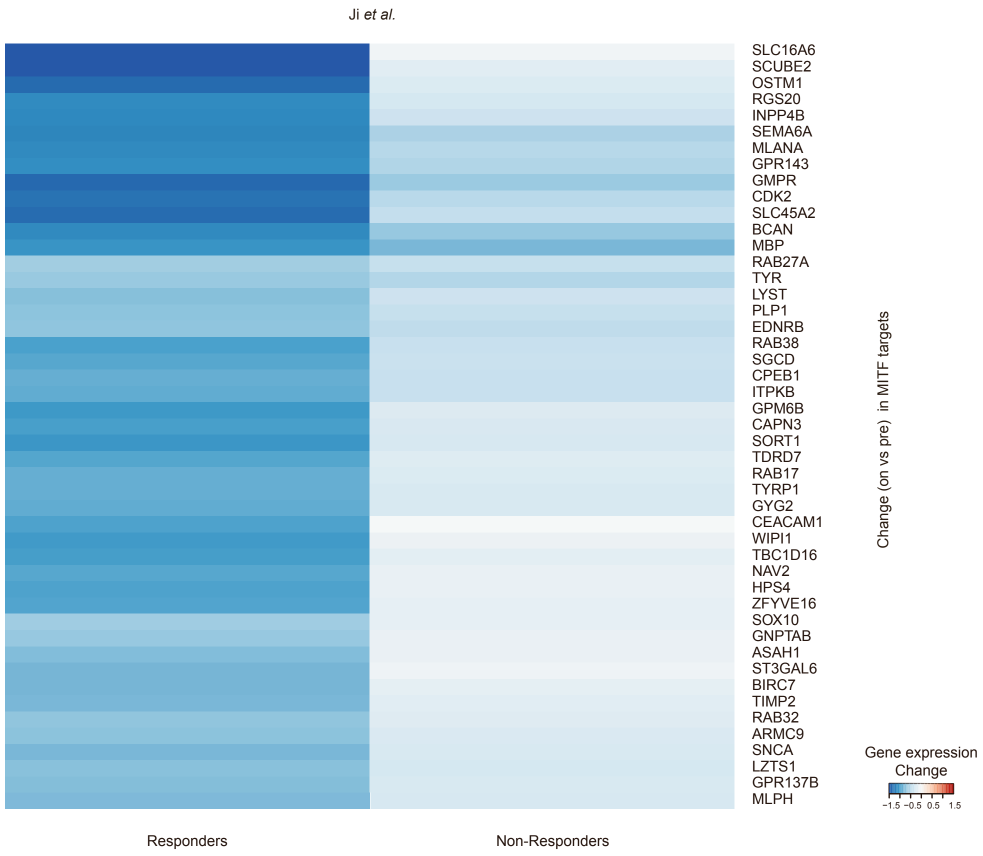

Figure S3 (related to Fig. 4). On treatment MITF downregulation predicts clinical outcome of anti-CTLA4-treated patients. Average change in gene expression upon treatment (pre- versus on-treatment) of MITF target genes in responders and non-responders to anti-CTLA-4 [S1].

References

[S1] Ji, R.R., Chasalow, S.D., Wang, L., Hamid, O., Schmidt, H., Cogswell, J., Alaparthy, S., Berman, D., Jure-Kunkel, M., Siemers, N.O., et al. (2012). An immune-active tumor microenvironment favors clinical response to ipilimumab. Cancer Immunology, Immunotherapy *61*, 1019–1031. 10.1007/s00262-011-1172-6.
