# Supplementary material for: An adverse tumor-protective effect of IDO1 inhibition
Source: Cell Rep Med. 2023 Feb 21;4(2):100941. doi: 10.1016/j.xcrm.2023.100941 (PMC9975322; doi:10.1016/j.xcrm.2023.100941)
Supplement: Document S2. Article plus supplemental information [file mmc2.pdf]

Cell Reports
Medicine

Article

An adverse tumor-protective effect of IDO1 inhibition

Graphical abstract

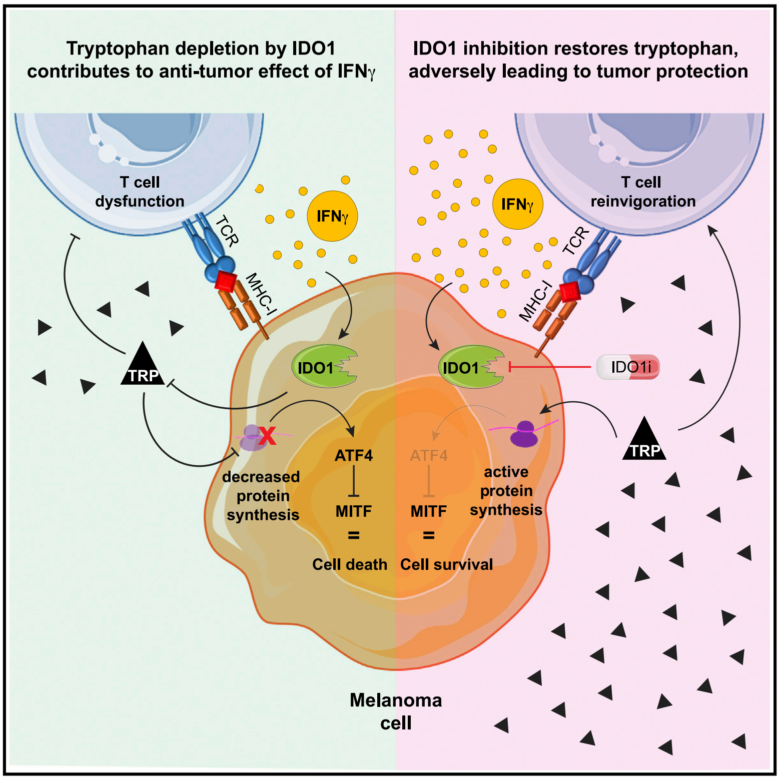

Authors

Juliana C.N. Kenski, Xinyao Huang, David W. Vredevoogd, ..., Jean-Christophe Marine, Reuven Agami, Daniel S. Peeper

Correspondence

d.peeper@nki.nl

In brief

Whereas IDO1 inhibitors were developed to reinvigorate T cells by restoring tryptophan, they lack clinical benefit. Kenski et al. discover an adverse effect of IDO1 inhibition: protecting melanoma cells from the effects of T cell-derived IFNγ. MITF plays a key role in this response *in vitro* and in patients.

Highlights

- Tryptophan depletion by IDO1 contributes to anti-tumor effect of IFNγ
- IFNγ shuts down general protein translation, which is reversed by IDO1 inhibition
- MITF downregulation contributes to melanoma T cell responses *in vitro* and in patients
- IDO1 inhibition prevents MITF downregulation, protecting melanoma cells from IFNγ

Kenski et al., 2023, Cell Reports Medicine *4*, 100941
February 21, 2023 © 2023 The Author(s).
https://doi.org/10.1016/j.xcrm.2023.100941

CellPress

Cell Reports Medicine

CellPress
OPEN ACCESS

Article

An adverse tumor-protective effect of IDO1 inhibition

Juliana C.N. Kenski,[1,13] Xinyao Huang,[1,13] David W. Vredevoogd,[1] Beaunelle de Bruijn,[1,6,7] Joleen J.H. Traets,[1] Sofía Ibáñez-Molero,[1] Sebastiaan M. Schieven,[1] Alex van Vliet,[1] Oscar Krijgsman,[1,8] Thomas Kuilman,[1,8] Joanna Pozniak,[4,5] Fabricio Loayza-Puch,[2,9] Alexandra M. Terry,[1,10] Judith Müller,[1,11] Meike E.W. Logtenberg,[1] Marjolein de Bruijn,[1] Pierre Levy,[1,12] Pierre-René Körner,[2] Colin R. Goding,[3] Ton N. Schumacher,[1] Jean-Christophe Marine,[4,5] Reuven Agami,[2] and Daniel S. Peeper[1,14,*]

[1]Division of Molecular Oncology and Immunology, Oncode Institute, the Netherlands Cancer Institute, Plesmanlaan 121, 1066 CX Amsterdam, the Netherlands
[2]Division of Oncogenomics, Oncode Institute, the Netherlands Cancer Institute, Plesmanlaan 121, 1066 CX Amsterdam, the Netherlands
[3]Ludwig Institute for Cancer Research, Nuffield Department of Medicine, University of Oxford, Old Road Campus, Headington, OX OX3 7DQ, UK
[4]Laboratory for Molecular Cancer Biology, Center for Cancer Biology, VIB, Leuven, Belgium
[5]Laboratory for Molecular Cancer Biology, Department of Oncology, KU Leuven, Leuven, Belgium
[6]Present address: Department of Oncology, KU Leuven, Leuven, Belgium
[7]Present address: VIB Center for Cancer Biology, KU Leuven, Leuven, Belgium
[8]Present address: Neogene Therapeutics, Amsterdam, the Netherlands
[9]Present address: German Cancer Research Center (DKFZ), Heidelberg, Germany
[10]Present address: Genmab, Utrecht, the Netherlands
[11]Present address: Siemens Healthineers, Cologne, Germany
[12]Present address: Vall d'Hebron Institute of Oncology, Barcelona, Spain
[13]These authors contributed equally
[14]Lead contact
*Correspondence: d.peeper@nki.nl
https://doi.org/10.1016/j.xcrm.2023.100941

SUMMARY

By restoring tryptophan, indoleamine 2,3-dioxygenase 1 (IDO1) inhibitors aim to reactivate anti-tumor T cells. However, a phase III trial assessing their clinical benefit failed, prompting us to revisit the role of IDO1 in tumor cells under T cell attack. We show here that IDO1 inhibition leads to an adverse protection of melanoma cells to T cell-derived interferon-gamma (IFNγ). RNA sequencing and ribosome profiling shows that IFNγ shuts down general protein translation, which is reversed by IDO1 inhibition. Impaired translation is accompanied by an amino acid deprivation-dependent stress response driving activating transcription factor-4 (ATF4)^{high}/microphtalmia-associated transcription factor (MITF)^{low} transcriptomic signatures, also in patient melanomas. Single-cell sequencing analysis reveals that MITF downregulation upon immune checkpoint blockade treatment predicts improved patient outcome. Conversely, MITF restoration in cultured melanoma cells causes T cell resistance. These results highlight the critical role of tryptophan and MITF in the melanoma response to T cell-derived IFNγ and uncover an unexpected negative consequence of IDO1 inhibition.

INTRODUCTION

Despite the profound improvement in melanoma outcome upon immune checkpoint blockade (ICB), therapy resistance limits clinical benefit for many patients.[1] This creates a need not only for uncovering additional targets for immunotherapy, but also for a better understanding of the mechanisms of action of their corresponding inhibitors. An example is indoleamine 2,3-dioxygenase 1 (IDO1), an enzyme induced by interferon-gamma (IFNγ)[2] and responsible for catalyzing the conversion of essential amino acid tryptophan to kynurenine.[3] Pre-clinical evidence suggested IDO1 as a key mechanism of acquired immune tolerance, by affecting several immune cells within the tumor microenvironment.[4,5] IDO1 expression and consequently low tryptophan levels increases intratumoral T regulatory cell (Treg) and myeloid-derived suppressor cell (MDSC) infiltration while decreasing dendritic cell (DC) antigen uptake, mediating IFN-γ-induced differentiation of monocytes into M2 macrophages and impairing cytotoxic T cell function.[5,6] Therefore, IDO1 inhibitors were developed and evaluated in combination with pembrolizumab (anti-programmed cell death-1/PD-1 antibody) in ECHO-301/KEYNOTE-252, a large phase 3 trial. Whereas the aforementioned pre-clinical evidence supported the use of IDO1 inhibition in the context of immunotherapy in solid tumors,[7,8] co-treatment of pembrolizumab with epacadostat failed to improve progression-free survival compared with

© 2023 The Author(s).
This is an open access article under the CC BY license (http://creativecommons.org/licenses/by/4.0/).

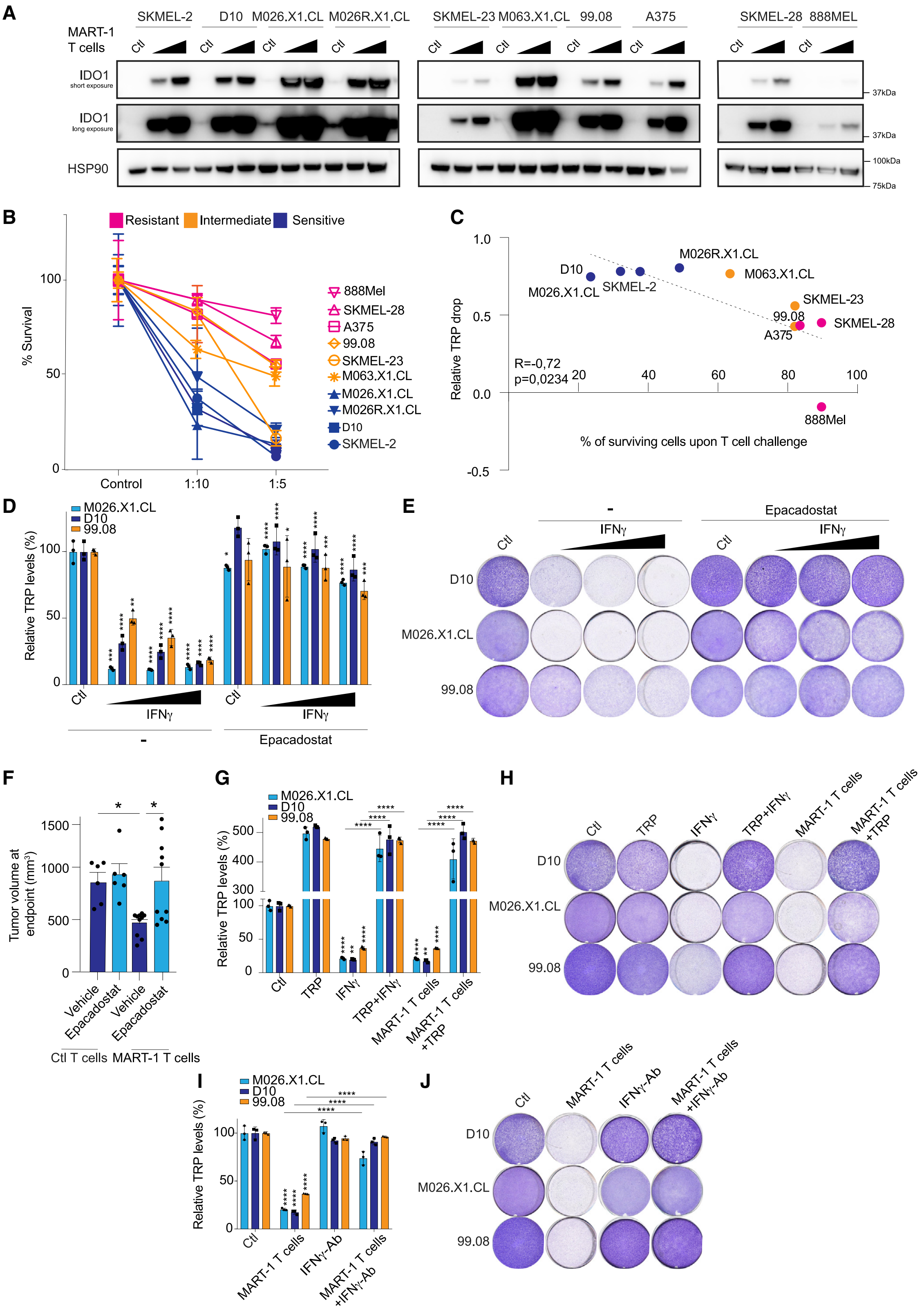

(legend on next page)

pembrolizumab alone[9]. These results led several pharmaceutical companies to scale down or terminate their IDO1 inhibitor studies.[10]

While several explanations were given for these disappointing results, including the potential low dose of epacadostat and selection bias, the reasons for the failure are still under debate.[10] Interestingly, whereas T cells are sensitive to tryptophan (TRP) deprivation in the tumor microenvironment (TME),[6,11] studies from the 80s and 90s suggested that, in fact, tumor cells, too, require TRP for viability.[12–14] These seemingly contradictory observations prompted us to study the functional interactions between IDO1, epacadostat, tumor cells, and T cells in more mechanistic detail.

RESULTS

TRP restoration by IDO1 inhibition protects tumor cells from T cell-mediated killing

To study under defined conditions the effect of IDO1 inhibition on tumor cells that are under T cell attack, we made use of a co-culture system that we previously established.[15] Briefly, we introduced the melanoma antigen recognized by T cell (MART-1)-specific T cell receptor (TCR; 1D3 clone) recognizing the human leukocyte antigen (HLA)-A2-restricted MART-1 peptide (amino acid [aa] 26–35) into $CD8^+$ T lymphocytes isolated from blood of healthy donors.[16] To ensure specific and equal recognition by MART-1 T cells and exclude potential confounding effects of differences in IFNγ signaling, antigen presentation machinery, and major histocompatibility complex (MHC) and antigen expression levels, we ectopically expressed through lentiviral transduction HLA-A*02:01 and MART-1 in a panel of human melanoma cell lines, including patient-derived melanoma xenograft (PDX) cell lines.[17] After confirming that the killing was TCRspecific (Figure S1A), we observed that exposure of these cell lines to the matched T cells led to common upregulation of IDO1 protein (albeit to varying degrees; Figure 1A). In parallel, we determined the relative susceptibility of this cell line panel to T cell-mediated killing. We observed a range of sensitivities, with some cell lines being highly sensitive, some showing an intermediate sensitivity, and others being relatively resistant (Figure 1B).

Because IDO1 activity leads to degradation of TRP in the TME, we measured the level of TRP in the culture medium after T cell treatment of melanoma cultures at a 1:10 T cell:tumor ratio, where we observed the biggest range of tumor sensitivities. The sensitivity of cell lines to T cell killing correlated significantly with the degree of TRP drop: the most sensitive cell lines had the highest relative decline in TRP levels after T cell challenge (Figure 1C), illustrating that melanoma cells can differentially suffer from low levels of TRP, while there may also be a contribution of the expression levels of IDO1 (Figure 1A).

We next asked whether the IDO1-induced TRP loss in fact contributed to the anti-tumor effect of IFNγ. As expected, treatment with IDO1 inhibitor epacadostat led to complete restoration of TRP levels (Figure 1D). More importantly, this was accompanied by a full rescue of the toxic effects of IFNγ (Figures 1E and S1B). IDO1 inhibition also rescued from T cell-induced killing (Figure S1C).

This result led us to investigate whether it could be recapitulated *in vivo* in a humanized adoptive cell transfer (ACT) setting. We used immunocompromised NOD/SCID IL2Rγ^{null} (NSG)

Figure 1. Tryptophan restoration by IDO1 inhibition protects tumor cells to T cell-mediated killing

Melanoma cells were co-cultured with MART-1 T cells (or no T cells as a control) at 1:5 and 1:10 T cell:tumor cell ratios for 24 h.

(A) After co-culture, cells were harvested and immunoblotted for IDO1 (short exposure and long exposure), all in parallel; HSP90 served as loading control.

(B) The same melanoma cell line panel was exposed to MART-1 T cells at indicated T cell:tumor cell ratios or no T cells as a control and stained with crystal violet after 6 days, and the percentage of surviving melanoma cells was quantified. Color coding indicates sensitivity to T cells: blue, relatively T cell-sensitive; orange, intermediate phenotype; pink, relatively resistant. Color coding was done arbitrarily for better visualization. The grouping of cell lines was not used for further analysis, and cell lines were always analyzed individually.

(C) Spearman correlation between relative (to control) tryptophan drop upon T cell exposure and percentage of surviving cells after T cell challenge, both at a 1:10 (T cell:tumor cell) ratio from the experiment shown in (B). Tryptophan (TRP) levels were measured from supernatant of melanoma and MART-1 T cell co-cultures by a fluorometric assay after 24 h of the experiment shown in (B).

(D) TRP concentrations from supernatants in (E) were measured by a fluorometric assay after 72 h of treatment. Statistical significance shown for the IFNγ-only group was tested comparing the IFNγ group against its control, whereas in the epacadostat-treated groups, it was compared with the corresponding IFNγ dose. The y axis shows normalized TRP levels compared with control.

(E) Cell lines were treated with IFNγ (2.5, 5, or 10 ng/mL) and/or epacadostat (2 μM), fixed and stained with crystal violet after 6 days. Quantification in Figure S1B.

(F) NSG mice received A375-MelanA cells subcutaneously into the flank, and after 3 days, 5 million MART-1-specific or control (untransduced) $CD8^+$ T cells were injected intravenously, and the mice were treated daily with epacadostat (100 mg/kg) orally. n = 6 mice for T cell control group and n = 10 mice for MART-1 T cell-treated group. Tumor sizes at endpoint are shown.

(G) TRP levels measured in supernatants of experiment shown in (H) after 72 h of treatment. Statistical testing of IFNγ and MART-1 T cell-only group was performed against their own control.

(H) D10, M026X1.CL, and 99.08 cells were treated with IFNγ (5 ng/mL), TRP (100 μg/mL), or MART-1 T cells (1:20 effector-to-target [E:T] ratio), fixed and stained after 6 days. Quantification in Figure S1G.

(I) Cell lines were co-cultured with MART-1 T cells at a 1:20 ratio in the presence or absence of IFNγ- blocking antibody. TRP levels were measured at the 72 h time point.

(J) Cells from experiment in (I) were fixed and stained with crystal violet after 6 days.

(G)–(J) belong to the same experiment and were separated for representation purposes. *In vitro* experiments were performed in two independent biological replicates, each in three technical replicates (available in Mendeley data [https://doi.org/10.17632/hd4h8fxdm9.1]). Bars represent ±SD for *in vitro* and ±SEM for *in vivo*. Statistical testing was done in the three technical replicates by one-way ANOVA with Tukey's post-hoc test. *p ≤ 0.05; **p ≤ 0.01; ***p ≤ 0.001; ****p ≤ 0.0001 and one-way ANOVA with Šidák's post-hoc for (F).

See also Figure S1.

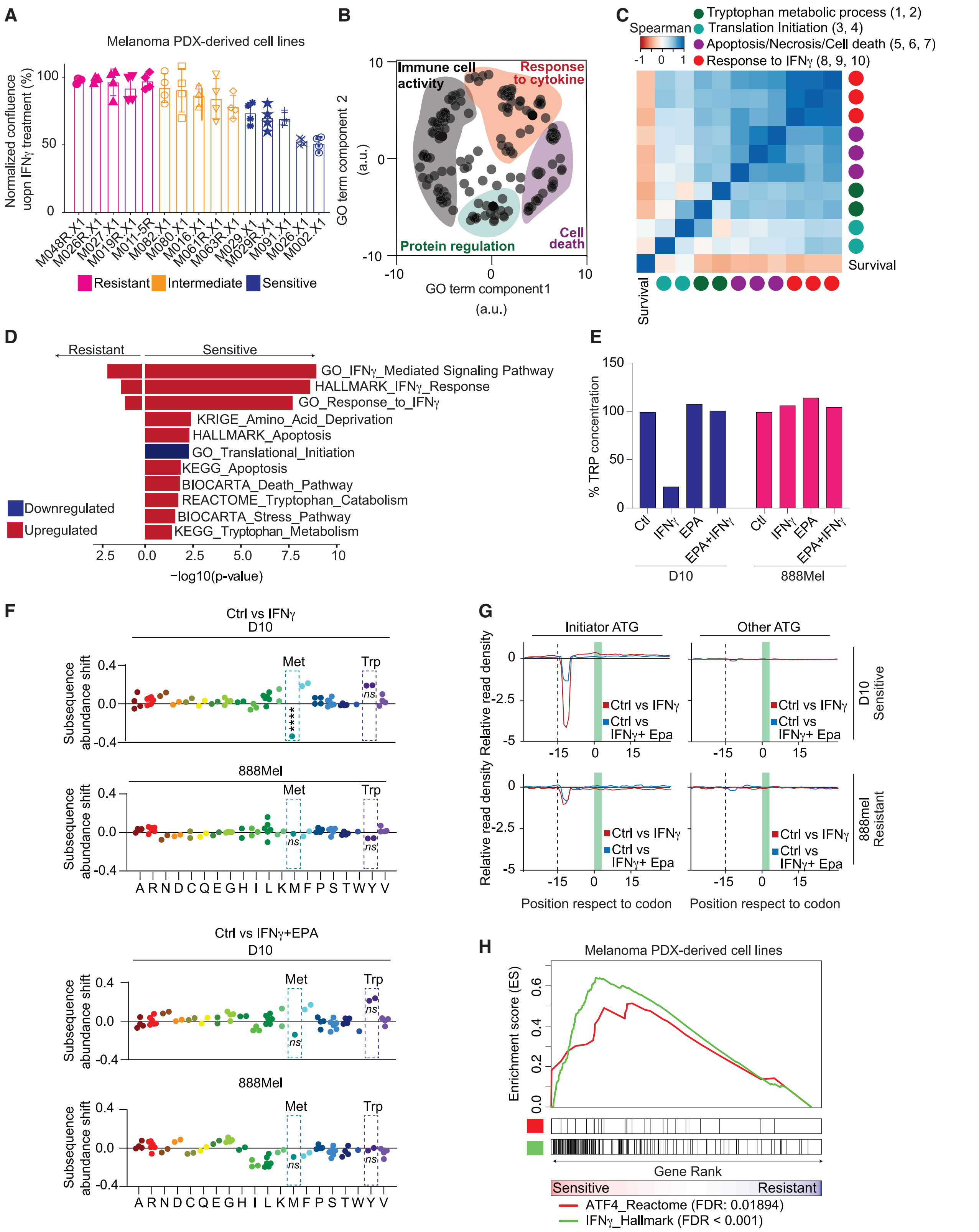

(legend on next page)

CellPress
OPEN ACCESS

mice and injected human melan-A antigen-expressing A375 melanoma cells subcutaneously. After 3 days, we injected either untransduced or MART-1 T cells via the tail vein and started the treatment with epacadostat. ACT of matched T cells caused significant tumor reduction (Figures 1F and S1D). However, recapitulating our *in vitro* data, we did not observe better tumor control upon epacadostat treatment *in vivo,* but instead a moderate, yet significant, increase in tumor expansion (Figures 1F and S1D). In an immunocompetent B16-OVA melanoma model, while we did not observe tumor acceleration upon IDO1 inhibition (likely because of simultaneous tumor cell and immune cell protection), there was again no improved tumor control (Figure S1E), in line with a previous study.[18] We confirmed by antibody depletion that CD8 T cells contributed to tumor control in this model (Figure S1F).

To determine whether this effect of epacadostat was mediated by TRP, we replenished this amino acid in the culture medium *in vitro*. TRP restoration, too, was able to revert the anti-tumor effect of either T cells or IFNγ treatment (Figures 1G, 1H, and S1G). This result is in concordance with the rescue in TRP levels caused by epacadostat treatment (Figure 1D) and indicate that the protection observed after IDO1 inhibition by this compound is indeed due to a specific TRP restoration. IFNγ was the major contributor of the T cell effect in this setting, because its blockade by a specific antibody significantly both rescued the decline in TRP and protected tumor cells (Figures 1I, 1J, and S1H). From these observations, we conclude that whereas IDO1 inhibitors were developed to reinvigorate immune cells in a TRP-deprived milieu, another consequence of TRP replenishment is that tumor cells are protected against T cell elimination or, in other words, an on-target adverse effect of IDO1 inhibition.

IFNγ-induced TRP depletion triggers general translation stalling associated with an activating transcription factor-4 (ATF4) stress response

To validate these findings in a more clinically relevant setting, we treated a panel of PDX melanoma cell lines with IFNγ and again observed intrinsic differences in their susceptibility to it (Figure 2A). To better understand the transcriptional reprogramming induced by this cytokine, we performed RNA sequencing of this PDX panel as a function of IFNγ treatment. Gene set enrichment analysis (GSEA) of their differentially expressed genes revealed enrichment of four distinct clusters of semantically related ontology terms: immune cell activity, protein regulation, response to cytokine, and cell death (Figure 2B).

These results prompted us to investigate whether these IFNγ-induced transcriptomic changes were related to each other and to IFNγ sensitivity. In line with our previous findings (Figure 1), we detected a strong correlation between gene sets related to TRP metabolic processes, IFNγ response-related pathways, cell death, and sensitivity (Figure 2C). Furthermore, pathway changes associated with a decrease in translation were also linked to higher expression of genes involved in TRP catabolism in this dataset (Figure 2C). Further exploring the differential response of sensitive and resistant cell lines to IFNγ, we observed that signatures for amino acid deprivation, TRP catabolism/metabolism, stress response, and death-related pathways were enriched in IFNγ-sensitive PDX cell lines (Figure 2D). This was also seen for a signature comprising translation initiation genes that are downregulated.

After demonstrating in PDX cell lines that IFNγ induces not only substantial transcriptional changes but also affects protein translation, we set out to confirm the latter observation in our original cell line panel (Figure 1) using ribosome profiling. We selected an IFNγ-responsive human melanoma cell line (D10), which showed a steep decline in TRP levels upon T cell and IFNγ co-culture (Figures 1C and 2E). As a control, we used an IFNγ-resistant cell line (888Mel), which failed to degrade TRP when encountering either IFNγ or T cells (Figures 1C and 2E). We recently demonstrated by differential ribosome codon reading (diricore) analysis that 48 h of IFNγ treatment leads to stalling at the TRP codon.[21] We show here that this is preceded by a reduced signal in the initiator ATG (first methionine) at position 12 at 20 h, when there are no significant changes yet in the

Figure 2. IFNγ-induced TRP depletion triggers general translation stalling associated with an ATF4 stress response

(A) A panel of patient-derived melanoma xenograft (PDX) cell lines was treated with 10 ng/mL IFNγ. Confluence was measured after 96 h by Incucyte live-cell imaging. Experiment was done with four technical replicates. Color coding was done arbitrarily for better visualization. Bars represent +/- SD.

(B) The same panel of PDX cell lines shown in (A) was analyzed by RNA sequencing (two biological replicates) after 24 h of IFNγ treatment (10 ng/mL). REVIGO[19] analysis was performed on significantly different Gene Ontology terms between IFNγ-treated and control samples.

(C) Heatmap shows Spearman correlation values between significantly different gene sets (IFNγ versus control) and survival (from A). Gene sets shown are the following (from left to right after survival): (1) GO_Negative_regulation_of_translation; (2) GO_Translational_initiation; (3) KEGG_Tryptophan_metabolism; (4) REACTOME_Tryptophan_catabolism, belonging to protein synthesis cluster; (5) HALLMARK_Apoptosis; (6) KEGG_Apoptosis; (7) BIOCARTA_Death_pathway, part of cell death cluster; (8) GO_Interferon_gamma_mediated_signaling; (9) HALLMARK_ Interferon_gamma_response; and (10) GO_Response_to_interferon_gamma, part of response to cytokine cluster.

(D) Gene set enrichment analysis was performed on the differentially expressed genes between control and IFNγ treatment in the IFNγ-sensitive (top 25% quartile) and IFNγ-resistant (top 25% quartile) cell lines from (A). All pathways shown have $p < 0.05$.

(E) Fluorometric TRP measurement in D10 and 888Mel cells used for ribosome profiling.

(F and G) Analysis of ribosome profiling performed after 20 h of IFNγ treatment (5 ng/mL) alone or in combination with epacadostat (2 μM). Control cells were left untreated.

(F) Ribosome accumulation at specific codons (dashed boxes). Ribosome accumulation at the start methionine codon in D10 cells after IFNγ treatment is a statistically significantly outlier when compared with ribosome occupancy of other codons.

(G) Panels representing differential ribosome occupancy determined by Diricore[20] analysis at the codons in proximity to the start site (initiator ATG; left panels) or other ATG codons (right panels) in control cells versus IFNγ-treated cells (red line) and IFNγ-treated cells versus cells treated with IFNγ in combination with epacadostat (blue line).

(H) Gene set enrichment analysis was performed in IFNγ-treated PDX cell lines comparing the IFNγ-sensitive (top 25% quartile) and IFNγ-resistant (top 25% quartile) cell lines.

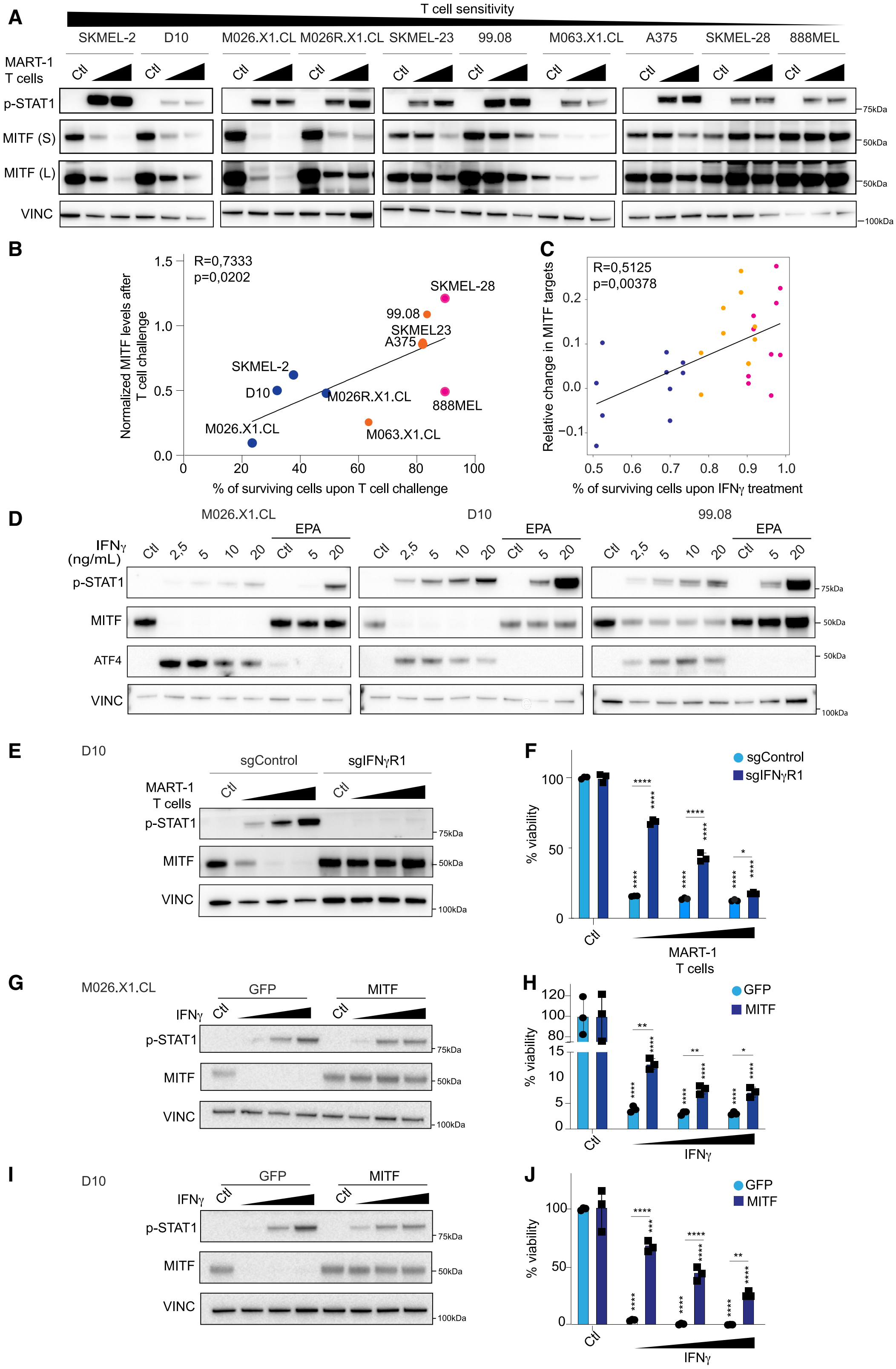

(legend on next page)

TRP codons (Figures 2F and 2G). A slower translation initiation rate with elongation proceeding at a normal pace (Figure 2G) causes an imbalance resulting in a significant change in ribosome occupancy at the ATG initiator, which indicates a strong general inhibition of translation,[20] here shown for IFNγ-treated D10 cells. This occurred when TRP levels were low due to IFNγ treatment (Figure 2E). Strikingly, co-treatment with epacadostat fully prevented protein translation shutdown (Figures 2F and 2G). Diminished translation also correlated with IFNγ responsiveness, because control IFNγ-resistant 888Mel cells failed to show this (Figures 2F and 2G).

A previous study has shown that cells under amino acid starvation stress can impair translation while selectively increasing translation of ATF4,[22] a key factor integrating protein translation and stress signaling, including amino acid deprivation.[23] To determine whether this was the case, GSEA was performed on the most IFNγ-sensitive vs. most IFNγ-resistant PDX cell lines. As expected, we observed an enrichment of IFNγ and ATF4 signatures in the sensitive cell lines (Figure 2H). Likely, the mechanism by which the sensing of uncharged tRNA-TRP in D10 cells treated with IFNγ occurs is via the general control nonderepressible-2 (GCN2), leading to eukaryotic initiation factor 2 (eIF2alpha) phosphorylation and ATF4 expression.[24,25] In cells not depleted for TRP (in 888Mel cells or D10 cells treated with epacadostat), there are no uncharged tRNA TRPs and thus no increase in ribosome accumulation at the start codons and, therefore, no ATF4-induced stress response.

MITF contributes to melanoma T cell sensitivity

ATF4 induction upon nutrient-deprivation stress has been associated with a reduction in the expression levels of microphtalmia-associated transcription factor (MITF), whereby the latter is transcriptionally suppressed by the first.[26] Furthermore, MITF is a critical survival factor for melanoma[27,28] and modulates the response to targeted tumor inhibitors.[29–31] These data, as well as the function of MITF in phenotypic plasticity and therapeutic resistance,[32] prompted us to investigate if it has a role in a tumor/T cell context. We observed distinct melanoma cell responses to T cell challenge: whereas several cell lines showed downregulation of MITF, others failed to diminish its expression (Figure 3A). The differential regulation of MITF was independent of activation of the early IFNγ signaling cascade because all examined cell lines expressed IFNγ receptors 1 and 2, as well as their downstream targets Janus kinase 1 and 2 (JAK1 and JAK2) and signal transducer and activator of transcription 1 (STAT1) (Figure S2A), while STAT1 was phosphorylated (Figure 3A).

This diversity in MITF response and its involvement in melanoma survival led us to explore any causal relationship with the observed differential susceptibility to T cell-induced cytotoxicity. We confirmed that the sensitivity of the tumor cells to T cells was strongly associated with their ability to downregulate MITF (Figure 3B). This correlation was also seen in the IFNγ-treated PDX cell line panel when analyzed by RNA sequencing (Figure 3C).

The inverse correlation between MITF expression and T cell susceptibility raised the possibility that the protection to IFNγ by IDO1 inhibition is due to the absence of an MITF downregulation. To investigate this, we co-treated melanoma cells with IFNγ and epacadostat. We observed that IDO1 inhibition prevented an ATF4-driven stress response and consequently MITF downregulation, even in the presence of active IFNγ signaling, as judged by STAT1 phosphorylation (Figure 3D). This result indicates that IFNγ-induced MITF regulation is a consequence of the modulation of endogenous TRP levels.

To determine whether the downregulation of MITF upon ribosome stalling was IFNγ dependent, we engineered IFNγ receptor 1 (IFNγR1) knockout clones and exposed them to T cells. After co-culture, MITF downregulation did not occur when IFNγ signaling was lacking (Figure 3E). These phenomena together resulted in a profound resistance to T cell cytotoxicity (Figure 3F).

To examine whether MITF plays a causal role, we prevented its downregulation in IFNγ-sensitive cells by introducing a cassette driving moderate expression of MITF. Because we used a heterologous promoter, MITF levels remained stable upon treatment with IFNγ (Figures 3G and 3I). The IFNγ signaling cascade was activated in both control and MITF-expressing cells, as

Figure 3. MITF contributes to melanoma T cell sensitivity

(A) Melanoma cell lines were co-cultured with T cells in 1:10 and 1:5 ratios for 24 h. Protein lysates were immunoblotted for MITF and phospho-STAT1 (Tyr701). Vinculin served as a loading control. Figure 1A and (A) belong to the same biological experiment, and western blotting was performed in parallel.

(B) Correlation between percentage of surviving cells after T cell challenge at a 1:10 (T cell:tumor cell) ratio and normalized (to loading control) change in MITF expression at the same ratio from immunoblot quantification data. Spearman correlation is plotted.

(C) Spearman correlation between relative survival upon IFNγ treatment in the PDX cell line panel from Figure 2A, plotted against MITF targets downregulation from RNA profiles after 24 h of IFNγ treatment.

For (B) and (C), color coding as in Figure 1B.

(D) Indicated cell lines were treated with IFNγ (2.5, 5, 10, and 20 ng/mL) or with epacadostat (2 μM) + 5 or 20 ng/mL IFNγ for 72 h and harvested for immunoblotting with the indicated antibodies, all in parallel. Vinculin served as a loading control.

(E and F) D10 cells carrying either single guide control (sgControl) or a sgRNA targeting IFNγR1 were co-cultured with T cells at 1:20/1:10/1:5 E:T ratios. Cells were harvested after 24 h for immunoblotting (E) or fixed and stained with crystal violet after 6 days, for which quantification is shown (F).

(G–J) D10 and M026.X1.CL cell lines were infected with lentivirus encoding GFP (as control) or MITF and were subsequently treated with IFNγ at 1, 5, or 10 ng/mL. Cells were either harvested after 72 h for immunoblotting (G and I) or fixed and stained with crystal violet after 6 days, for which quantification is shown (H and J).

Experiments were performed in at least two independent biological replicates, each in three technical replicates (available in Mendeley data [https://doi.org/10.17632/hd4h8fxdm9.1]).

Statistical testing comparing either IFNγ treatment or T cell treatment against controls was done with one-way ANOVA and Dunnet's post-hoc. Comparisons between sgControl versus sgIFNγR1 or GFP versus MITF were done by unpaired Student's t test (two-tailed). Bars represent +/-SD. *$p \leq 0.05$; **$p \leq 0.01$; ***$p \leq 0.001$; ****$p \leq 0.0001$.

See also Figure S2.

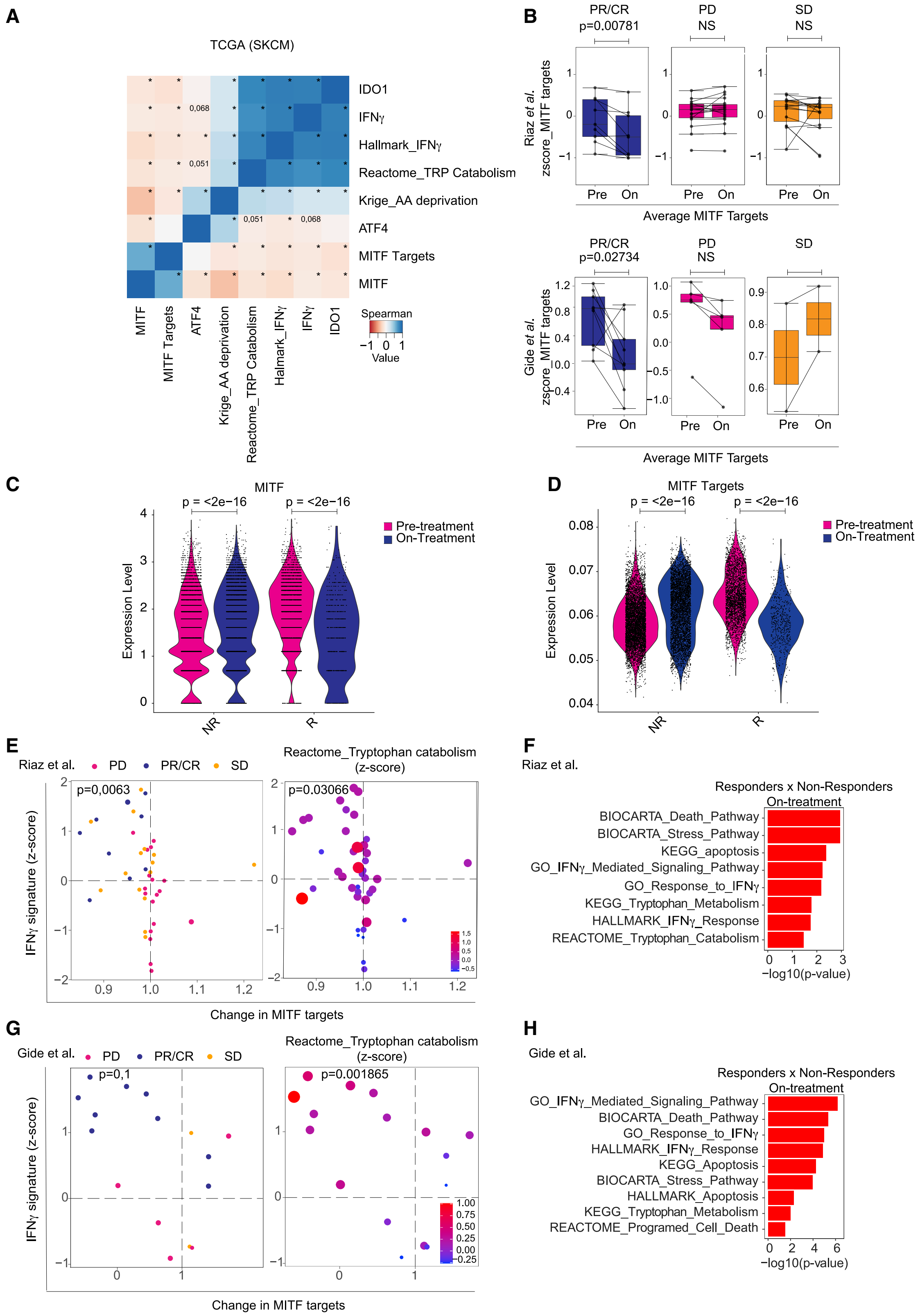

(legend on next page)

confirmed by STAT1 phosphorylation (Figures 3G and 3I). Importantly, the enforced inability of (patient-derived) melanoma cells to downregulate MITF diminished their sensitivity to IFNγ (Figures 3H and 3J). These data demonstrate that the ability of melanoma cells to downregulate MITF is essential for their intrinsic susceptibility to the anti-tumor effect of IFNγ.

On-treatment MITF downregulation predicts clinical outcome of ICB

To determine whether these results bear clinical relevance, we analyzed MITF expression as well as its target genes in The Cancer Genome Atlas (TCGA) melanoma cohort. This revealed that MITF expression, together with that of its transcriptional targets, is inversely correlated with IFNγ itself, an IFNγ signature, and the IFNγ-inducible gene IDO1. This inverse correlation was also seen for ATF4, amino acid deprivation, and TRP catabolism genetic signatures (Figure 4A), in accordance with our previous observations in cell lines (Figure 2C).

Since T cells can be reinvigorated and triggered to produce IFNγ by ICB, we subsequently analyzed two independent gene expression datasets of anti-PD-1-treated patients for whom both pre- and on-treatment samples were available.[34,35] In line with our *in vitro* data, those patient tumors that responded to immunotherapy downregulated MITF target genes on treatment in both cohorts, whereas the non-responding tumors did not (Figure 4B). Similar results were observed for anti-cytotoxic T lymphocyte-associated protein 4 (CTLA-4)-treated patients[38] (Figure S3A). We confirmed these findings in patient cohorts analyzed at the single-cell level.[36] We observed that melanoma cells in patients responding to treatment had significantly lower levels of MITF and MITF target genes than prior to treatment or non-responders (Figures 4C and 4D). Additional mining of the Riaz[35] and Guide[34] datasets revealed that melanomas with a high IFNγ signature[37] and the strongest change in MITF are most likely to respond to anti-PD-1 (Figures 4E and 4G, left panels). Responding tumors also showed significantly higher expression levels of genes related to TRP catabolism than the other samples (Figures 4E and 4G, right panels). Furthermore, stress response, IFNγ signaling, and TRP metabolism/catabolism apoptosis-related gene sets were enriched on treatment in responders to anti-PD-1 treatment (Figures 4F and 4H). Together, these results demonstrate that the intrinsic sensitivity of melanoma cells to IFNγ, T cells, and PD-1 blockade correlates with their ability to show a dynamic MITF response.

DISCUSSION

We uncovered an unexpected, and undesired, on-target effect of IDO1 inhibition: whereas IDO1 inhibitors were developed to protect cytotoxic T cells and other immune cells against the deleterious effects of TRP depletion in the TME, we demonstrate here that IDO1 inhibition (or TRP replenishment) also leads to protection of melanoma cells from T cell elimination *in vitro* and *in vivo*. Given that IFNγ can exert a strong bystander effect, influencing not just antigen-positive cells,[39] the protection by epacadostat may extend to remote tumor cells. While we do not wish to claim that this is a major cause for the failure of the ECHO-301 IDO1 trial,[9,40] our results do shed light on a critical aspect of IDO1 inhibition that was not previously appreciated. This may be an opportunity to guide the design of any future immunotherapy application of IDO1 inhibitors.

Our data suggest that targeting MITF could be a promising approach in combination with immunotherapy, since its downregulation in melanoma cells under T cell attack contributes to their propensity to be eliminated. MITF is an important survival factor for melanoma, and changes in its expression levels can have major consequences in several contexts.[28,32] However, to study this *in vitro* is challenging, as was noted by us and many other groups: melanocytes and melanoma cells do not tolerate strong modulations of the expression levels of MITF whether by depletion or overexpression.[32,41–44] This notwithstanding, our data are consistent with the notion that MITF is a key regulator of differential cell states when cells experience various types of stress.[32,45,46] Additionally, chronic exposure of melanoma cells to T cells can lead to de-differentiation and resistance.[47] Taken together, our data suggest that patients with immunotherapy-refractory melanoma could benefit from an acute therapy-induced decrease of MITF levels to increase their susceptibility to cytotoxic T cells.

The clinical benefit of epacadostat was investigated in the context of anti-PD-1.[9] This is in keeping with the increasing awareness that for most advanced cancers, therapy resistance

Figure 4. On-treatment MITF downregulation predicts clinical outcome of immune checkpoint blockade

(A) Heatmap showing Spearman correlations between *IDO1*, *MITF*, and *IFNγ* gene expression, average MITF targets, mean of amino acid deprivation signature,[33] and average of Hallmark IFNγ and Reactome TRP catabolism signatures from SKCM melanoma cohort from TCGA. *$p \leq 0.05$.

(B) Average change in gene expression of MITF target genes comparing pre-treatment and on-treatment samples for partial responders (PRs), complete responders (CRs), stable disease (SD), and progressive disease (PD) patients for anti-PD-1 treatment, for two clinical cohorts.[34,35] Box plots represent the median and 1.5 interquartile range (IQR) of the upper quartile/lower quartile. Bars represent +/- SD.

(C and D) Single-cell RNA sequencing data from pre- and on-immune checkpoint blockade-treated patients[36] were analyzed for MITF and MITF targets changes. Each dot represents a cell. p values calculated by Wilcoxon signed-rank test.

(E and G) The average expression level of the IFNγ signature[33] on treatment was plotted against the change in MITF target gene expression (ratio between normalized counts on versus pre-treatment). Left panels show clinical response data (color code at the top). Statistical analysis was performed by χ-squared test comparing top left quadrants with the remainder quadrants. Right panels show expression of the gene set (Reactome) TRP catabolism. Size and color of the circles indicate effect size of pathway enrichment. Enrichment values in the top quadrants (MITF target gene downregulation/IFNγ signature high) were significantly different from the other three quadrants (Mann-Whitney U ranked, p value indicated).

(F and H) Gene set enrichment analysis (GSEA) performed on on-treatment samples comparing responders (PRs/CRs) and non-responders (PD/SD). All pathways shown have $p < 0.05$.

(B, top panel, E, and F) Riaz clinical cohort.[35] (B, bottom panel, G, and H) Gide clinical cohort.[34]

See also Figure S3.

limits the benefit of single-agent therapies. Therefore, thousands of clinical trials are currently testing combination treatments, often with anti-PD-1 (or variations thereof). As we recently argued, however, with the increasing numbers of (immuno)therapeutics developed, the possibilities for combination treatments dramatically outnumber the patients available to enroll in clinical trials.[48] Rational design, informed by fundamental biological and mechanistic insight, will be required to solve this clinical problem. The current study provides an example of how a better mechanistic understanding may contribute to this in that it not only uncovers an on-target adverse effect of IDO1 inhibition but also raises the possibility that pharmacologic MITF intervention might be explored to improve immunotherapy outcome of patients with melanoma.

Limitations of the study

This study has a number of limitations that should be considered. This includes the technical challenge of manipulating MITF in a stable and reliable manner to provide genetic confirmation of some of our findings. Similarly, while our data suggest that targeting MITF might be beneficial in combination with IDO1 inhibition, the lack of a specific MITF inhibitor precludes pharmacologic testing. Furthermore, our findings may be particularly relevant to IFNγ-rich tumors and MITF-expressing melanomas. More complex immunocompetent models will be required for formal testing of our hypothesis *in vivo* that the positive effect that IDO1 inhibition may have on CD8 T cells is counteracted by the tumor protection described here. Lastly, datasets from patient melanomas with acquired ICB resistance and IDO1 inhibition are scarce, hindering such clinical corroboration.

STAR★METHODS

Detailed methods are provided in the online version of this paper and include the following:

- KEY RESOURCES TABLE
- RESOURCE AVAILABILITY
  - Lead contact
  - Materials availability
  - Data and code availability
- EXPERIMENTAL MODEL AND SUBJECT DETAILS
  - Human primary CD8$^+$ T cells
  - Cell lines and culture conditions
  - Animal studies
- METHOD DETAILS
  - Lentivirus production
  - Cytotoxicity assays
  - Immunoblotting and antibodies
  - RNA-sequencing and analysis
  - Ribosome profiling and analysis
  - Tryptophan measurement
  - Bioinformatic analysis
- QUANTIFICATION AND STATISTICAL ANALYSIS
  - Statistical testing
- ADDITIONAL RESOURCES
  - Illustration

SUPPLEMENTAL INFORMATION

Supplemental information can be found online at https://doi.org/10.1016/j.xcrm.2023.100941.

ACKNOWLEDGMENTS

We thank all members of the Peeper and Schumacher laboratories for helpful advice, and especially Susan van Hal for technical help during revision. We are also grateful to Riccardo Mezzadra and Raquel Gomez-Eerland for contributing to the setup of the melanoma:T cell co-culture system and Olaf van Tellingen for helping with *in vivo* drug formulation. We thank Georgina Long for helpful discussions. This work was financially supported by the European Research Council under the European Union's Seventh Framework Programme (FP7/2007–2013)/ERC synergy (grant agreement 319661 COMBATCANCER to D.S.P.), Science Without Borders Program-CAPES (J.C.N.K., grant #1334713-6), a Queen Wilhelmina Award by the Dutch Cancer Society (D.S.P.), and base funding by Oncode Institute (D.S.P.).

AUTHOR CONTRIBUTIONS

X.H., J.C.N.K., D.W.V., J..M., and D.S.P. conceived the study. J.C.N.K. carried out all experiments for this manuscript, with contributions of X.H. to Figures 2, 3C, and 3D and to reaching preliminary conclusions. A.M.T. contributed to Figures 1D, 1E, 3D, and 3G–3J. M.E.W. L. and M.d.B. contributed to characterizing the PDX cell lines used for Figure 2 and for PDX RNA sequencing. X.H., S.I.-M., and T.N.S. contributed to establishing the tumor-T cell system; X.H. also contributed to system optimization. B.d.B. and J.C.N.K. performed the *in vivo* experiments. S.M.S. and S.I.-M. performed experiments for revision. J.J.H.T., A.v.V., T.K., and O.K. performed bioinformatics analyses. P.L., F.L.-P., P.-R.K., and R.A. performed and analyzed Ribo-Seq, with a contribution from X.H. and J.C.N.K. J.P. and J.-C.M. generated and analyzed single-cell sequencing data. C.R.G. provided critical input on the analysis of amino acid stress signaling, and J.C.N.K. and D.S.P. wrote the manuscript. The project was supervised by D.S.P. All authors reviewed and approved the manuscript.

DECLARATION OF INTERESTS

D.S.P. is co-founder, shareholder, and advisor of Immagene, which is unrelated to this study.

Received: April 1, 2022
Revised: November 19, 2022
Accepted: January 20, 2023
Published: February 21, 2023

REFERENCES

1. Sharma, P., Hu-Lieskovan, S., Wargo, J.A., and Ribas, A. (2017). Primary, adaptive, and acquired resistance to cancer immunotherapy. Cell *168*, 707–723. https://doi.org/10.1016/j.cell.2017.01.017.
2. Yoshida, R., Imanishi, J., Oku, T., Kishida, T., and Hayaishi, O. (1981). Induction of pulmonary indoleamine 2,3-dioxygenase by interferon. Proc. Natl. Acad. Sci. USA *78*, 129–132. https://doi.org/10.1073/pnas.78.1.129.
3. Kotake, Y., and Masayama, I. (1936). Studien über den intermediären Stoffwechsel des Tryptophans XVIII—XXIV. Hoppe-Seyler's. Zeitschrift für. physiologische Chemie *243*, 237–265. https://doi.org/10.1515/bchm2.1936.243.6.237.
4. Uyttenhove, C., Pilotte, L., Théate, I., Stroobant, V., Colau, D., Parmentier, N., Boon, T., and van den Eynde, B.J. (2003). Evidence for a tumoral immune resistance mechanism based on tryptophan degradation by indoleamine 2,3-dioxygenase. Nat. Med. *9*, 1269–1274. https://doi.org/10.1038/nm934.

CellPress
OPEN ACCESS

5. Meireson, A., Devos, M., and Brochez, L. (2020). Ido expression in cancer: different compartment, different functionality? Front. Immunol. *11*, 531491. https://doi.org/10.3389/fimmu.2020.531491.

6. Munn, D.H., Shafizadeh, E., Attwood, J.T., Bondarev, I., Pashine, A., and Mellor, A.L. (1999). Inhibition of T cell proliferation by macrophage tryptophan catabolism. J. Exp. Med. *189*, 1363–1372. https://doi.org/10.1084/jem.189.9.1363.

7. Wainwright, D.A., Chang, A.L., Dey, M., Balyasnikova, I.v., Kim, C.K., Tobias, A., Cheng, Y., Kim, J.W., Qiao, J., Zhang, L., et al. (2014). Durable therapeutic efficacy utilizing combinatorial blockade against Ido, CTLA-4, and PD-L1 in mice with brain tumors. Clin. Cancer Res. *20*, 5290–5301. https://doi.org/10.1158/1078-0432.CCR-14-0514.

8. Spranger, S., Koblish, H.K., Horton, B., Scherle, P.A., Newton, R., and Gajewski, T.F. (2014). Mechanism of tumor rejection with doublets of CTLA-4, PD-1/PD-L1, or Ido blockade involves restored IL-2 production and proliferation of CD8+ T cells directly within the tumor microenvironment. J. Immunother. Cancer *2*, 3–14. https://doi.org/10.1186/2051-1426-2-3.

9. Long, G.v., Dummer, R., Hamid, O., Gajewski, T.F., Caglevic, C., Dalle, S., Arance, A., Carlino, M.S., Grob, J.J., Kim, T.M., et al. (2019). Epacadostat plus pembrolizumab versus placebo plus pembrolizumab in patients with unresectable or metastatic melanoma (ECHO-301/KEYNOTE-252): a phase 3, randomised, double-blind study. Lancet Oncol. *20*, 30274–30278. https://doi.org/10.1016/S1470-2045.

10. (2018). Companies scaling back Ido1 inhibitor trials. Cancer Discov. *8*. https://doi.org/10.1158/2159-8290.CD-ND2018-007.

11. Munn, D.H., Zhou, M., Attwood, J.T., Bondarev, I., Conway, S.J., Marshall, B., Brown, C., and Mellor, A.L. (1998). Prevention of allogeneic fetal rejection by tryptophan catabolism. Science *281*, 1191–1193. https://doi.org/10.1126/science.281.5380.1191.

12. Burke, F., Knowles, R.G., East, N., and Balkwill, F.R. (1995). THE role of indoleamine 2,3-dioxygenase in the anti-tumour activity of human interferon-γ in vivo. Int. J. Cancer *60*, 115–122. https://doi.org/10.1002/ijc.2910600117.

13. Takikawa, O., Kuroiwa, T., Yamazaki, F., and Kido, R. (1988). Mechanism of interferon-γ action. Characterization of indoleamine 2,3-dioxygenase in cultured human cells induced by interferon -γ and evaluation of the enzyme-mediated tryptophan degradation in its anticellular activity. J. Biol. Chem. *263*, 2041–2048.

14. Ozaki, Y., Edelstein, M.P., and Duch, D.S. (1988). Induction of indoleamine 2,3-dioxygenase: a mechanism of the antitumor activity of interferon γ. Proc. Natl. Acad. Sci. USA *85*, 1242–1246. https://doi.org/10.1073/pnas.85.4.1242.

15. Vredevoogd, D.W., Kuilman, T., Ligtenberg, M.A., Boshuizen, J., Stecker, K.E., de Bruijn, B., Krijgsman, O., Huang, X., Kenski, J.C.N., Lacroix, R., et al. (2019). Augmenting immunotherapy impact by lowering tumor TNF cytotoxicity threshold. Cell *178*, 585–599.e15. https://doi.org/10.1016/j.cell.2019.06.014.

16. Gomez-Eerland, R., Nuijen, B., Heemskerk, B., van Rooij, N., van den Berg, J.H., Beijnen, J.H., Uckert, W., Kvistborg, P., Schumacher, T.N., Haanen, J.B.A.G., and Jorritsma, A. (2014). Manufacture of gene-modified human T-cells with a memory stem/central memory phenotype. Hum. Gene Ther. Methods *25*, 277–287. https://doi.org/10.1089/hgtb.2014.004.

17. Kemper, K., Krijgsman, O., Kong, X., Cornelissen-Steijger, P., Shahrabi, A., Weeber, F., van der Velden, D.L., Bleijerveld, O.B., Kuilman, T., Kluin, R.J.C., et al. (2016). BRAFV600E kinase domain duplication identified in therapy-refractory melanoma patient-derived xenografts. Cell Rep. *16*, 263–277. https://doi.org/10.1016/j.celrep.2016.05.064.

18. Georganaki, M., Ramachandran, M., Tuit, S., Núñez, N.G., Karampatzakis, A., Fotaki, G., van Hooren, L., Huang, H., Lugano, R., Ulas, T., et al. (2020). Tumor endothelial cell up-regulation of Ido1 is an immunosuppressive feed-back mechanism that reduces the response to CD40-stimulating immunotherapy. OncoImmunology *9*, 1730538. https://doi.org/10.1080/2162402X.2020.1730538.

19. Supek, F., Bošnjak, M., Škunca, N., and Šmuc, T. (2011). Summarizes and visualizes long lists of gene ontology terms. PLoS One *6*, 21800. https://doi.org/10.1371/journal.pone.0021800.

20. Loayza-Puch, F., Rooijers, K., Buil, L.C.M., Zijlstra, J., Oude Vrielink, J.F., Lopes, R., Ugalde, A.P., van Breugel, P., Hofland, I., Wesseling, J., et al. (2016). Tumour-specific proline vulnerability uncovered by differential ribosome codon reading. Nature *530*, 490–494. https://doi.org/10.1038/nature16982.

21. Bartok, O., Pataskar, A., Nagel, R., Laos, M., Goldfarb, E., Hayoun, D., Levy, R., Körner, P.R., Kreuger, I.Z.M., Champagne, J., et al. (2021). Anti-tumour immunity induces aberrant peptide presentation in melanoma. Nature *590*, 332–337. https://doi.org/10.1038/s41586-020-03054-1.

22. Harding, H.P., Novoa, I., Zhang, Y., Zeng, H., Wek, R., Schapira, M., and Ron, D. (2000). Regulated translation initiation controls stress-induced gene expression in mammalian cells. Mol. Cell *6*, 1099–1108. https://doi.org/10.1016/s1097-2765(00)00108-8.

23. Pakos-Zebrucka, K., Koryga, I., Mnich, K., Ljujic, M., Samali, A., and Gorman, A.M. (2016). The integrated stress response. EMBO Rep. *17*, 1374–1395. https://doi.org/10.15252/embr.201642195.

24. Dong, J., Qiu, H., Garcia-Barrio, M., Anderson, J., and Hinnebusch, A.G. (2000). Uncharged tRNA activates GCN2 by displacing the protein kinase moiety from a bipartite tRNA-binding domain. Mol. Cell *6*, 269–279. https://doi.org/10.1016/S1097-2765(00)00028-9.

25. Ye, J., Kumanova, M., Hart, L.S., Sloane, K., Zhang, H., de Panis, D.N., Bobrovnikova-Marjon, E., Diehl, J.A., Ron, D., and Koumenis, C. (2010). The GCN2-ATF4 pathway is critical for tumour cell survival and proliferation in response to nutrient deprivation. EMBO J. *29*, 2082–2096. https://doi.org/10.1038/emboj.2010.81.

26. Falletta, P., Sanchez-del-Campo, L., Chauhan, J., Effern, M., Kenyon, A., Kershaw, C.J., Siddaway, R., Lisle, R., Freter, R., Daniels, M.J., et al. (2017). Translation reprogramming is an evolutionarily conserved driver of phenotypic plasticity and therapeutic resistance in melanoma. Genes Dev. *31*, 18–33. https://doi.org/10.1101/gad.290940.116.

27. Tsao, H., Chin, L., Garraway, L.A., and Fisher, D.E. (2012). Melanoma: from mutations to medicine. Genes Dev. *26*, 1131–1155. https://doi.org/10.1101/gad.191999.112.

28. Garraway, L.A., Widlund, H.R., Rubin, M.A., Getz, G., Berger, A.J., Ramaswamy, S., Beroukhim, R., Milner, D.A., Granter, S.R., Du, J., et al. (2005). Integrative genomic analyses identify MITF as a lineage survival oncogene amplified in malignant melanoma. Nature *436*, 117–122. https://doi.org/10.1038/nature03664.

29. Konieczkowski, D.J., Johannessen, C.M., Abudayyeh, O., Kim, J.W., Cooper, Z.A., Piris, A., Frederick, D.T., Barzily-Rokni, M., Straussman, R., Haq, R., et al. (2014). A melanoma cell state distinction influences sensitivity to MAPK pathway inhibitors. Cancer Discov. *4*, 816–827. https://doi.org/10.1158/2159-8290.CD-13-0424.

30. Müller, J., Krijgsman, O., Tsoi, J., Robert, L., Hugo, W., Song, C., Kong, X., Possik, P.A., Cornelissen-Steijger, P.D.M., Geukes Foppen, M.H., et al. (2014). Low MITF/AXL ratio predicts early resistance to multiple targeted drugs in melanoma. Nat. Commun. *5*, 5712. https://doi.org/10.1038/ncomms6712.

31. Smith, M.P., Sanchez-Laorden, B., O'brien, K., Brunton, H., Ferguson, J., Young, H., Dhomen, N., Flaherty, K.T., Frederick, D.T., Cooper, Z.A., et al. (2014). The immune microenvironment confers resistance to MAPK pathway inhibitors through macrophage-derived TNF. Cancer Discov. *4*, 1214–1229. https://doi.org/10.1158/2159-8290.CD-13-1007.

32. Goding, C.R., and Arnheiter, H. (2019). Mitf—the first 25 years. Genes Dev. *33*, 983–1007. https://doi.org/10.1101/gad.324657.119.

33. Krige, D., Needham, L.A., Bawden, L.J., Flores, N., Farmer, H., Miles, L.E.C., Stone, E., Callaghan, J., Chandler, S., Clark, V.L., et al. (2008). CHR-2797: an antiproliferative aminopeptidase inhibitor that leads to amino acid deprivation in human leukemic cells. Cancer Res. *68*, 6669–6679. https://doi.org/10.1158/0008-5472.CAN-07-6627.

34. Gide, T.N., Quek, C., Menzies, A.M., Tasker, A.T., Shang, P., Holst, J., Madore, J., Lim, S.Y., Velickovic, R., Wongchenko, M., et al. (2019). Distinct immune cell populations define response to anti-PD-1 monotherapy and anti-PD-1/anti-CTLA-4 combined therapy. Cancer Cell *35*, 238–255.e6. https://doi.org/10.1016/j.ccell.2019.01.003.

35. Riaz, N., Havel, J.J., Makarov, V., Desrichard, A., Urba, W.J., Sims, J.S., Hodi, F.S., Martín-Algarra, S., Mandal, R., Sharfman, W.H., et al. (2017). Tumor and microenvironment evolution during immunotherapy with nivolumab. Cell *171*, 934–949.e16. https://doi.org/10.1016/j.cell.2017.09.028.

36. Pozniak, J., Pedri, D., Landeloos, E., van Herck, Y., Antoranz, A., Karras, P., Nowosad, A., Makhzami, S., Bervoets, G., Dewaele, M., et al. (2022). A TCF4/BRD4-dependent regulatory network confers cross-resistance to targeted and immune checkpoint therapy in melanoma. Preprint at bioRxiv. https://doi.org/10.1101/2022.08.11.502598.

37. Ayers, M., Lunceford, J., Nebozhyn, M., Murphy, E., Loboda, A., Kaufman, D.R., Albright, A., Cheng, J.D., Kang, S.P., Shankaran, V., et al. (2017). IFN-γ-related mRNA profile predicts clinical response to PD-1 blockade. J. Clin. Invest. *127*, 2930–2940. https://doi.org/10.1172/JCI91190.

38. Ji, R.R., Chasalow, S.D., Wang, L., Hamid, O., Schmidt, H., Cogswell, J., Alaparthy, S., Berman, D., Jure-Kunkel, M., Siemers, N.O., et al. (2012). An immune-active tumor microenvironment favors clinical response to ipilimumab. Cancer Immunol. Immunother. *61*, 1019–1031. https://doi.org/10.1007/s00262-011-1172-6.

39. Hoekstra, M.E., Bornes, L., Dijkgraaf, F.E., Philips, D., Pardieck, I.N., Toebes, M., Thommen, D.S., van Rheenen, J., and Schumacher, T.N.M. (2020). Long-distance modulation of bystander tumor cells by CD8+ T-cell-secreted IFN-γ. Nat. Can. (Que.) *1*, 291–301. https://doi.org/10.1038/s43018-020-0036-4.

40. Muller, A.J., Manfredi, M.G., Zakharia, Y., and Prendergast, G.C. (2019). Inhibiting Ido pathways to treat cancer: lessons from the ECHO-301 trial and beyond. Semin. Immunopathol. *41*, 41–48. https://doi.org/10.1007/s00281-018-0702-0.

41. Wellbrock, C., and Marais, R. (2005). Elevated expression of MITF counteracts B-RAF-stimulated melanocyte and melanoma cell proliferation. J. Cell Biol. *170*, 703–708. https://doi.org/10.1083/jcb.200505059.

42. Selzer, E., Wacheck, V., Lucas, T., Heere-Ress, E., Wu, M., Weilbaecher, K.N., Schlegel, W., Valent, P., Wrba, F., Pehamberger, H., et al. (2002). The melanocyte-specific isoform of the microphthalmia transcription factor affects the phenotype of human melanoma. Cancer Res. *62*, 2098–2103.

43. Carreira, S., Goodall, J., Denat, L., Rodriguez, M., Nuciforo, P., Hoek, K.S., Testori, A., Larue, L., and Goding, C.R. (2006). Mitf regulation of Dia1 controls melanoma proliferation and invasiveness. Genes Dev. *20*, 3426–3439. https://doi.org/10.1101/gad.406406.

44. Loercher, A.E., Tank, E.M.H., Delston, R.B., and Harbour, J.W. (2005). MITF links differentiation with cell cycle arrest in melanocytes by transcriptional activation of INK4A. J. Cell Biol. *168*, 35–40. https://doi.org/10.1083/jcb.200410115.

45. Cheli, Y., Ohanna, M., Ballotti, R., and Bertolotto, C. (2010). Fifteen-year quest for microphthalmia-associated transcription factor target genes. Pigment Cell Melanoma Res. *23*, 27–40. https://doi.org/10.1111/j.1755-148X.2009.00653.x.

46. Hoek, K.S., and Goding, C.R. (2010). Cancer stem cells versus phenotype-switching in melanoma. Pigment Cell Melanoma Res. *23*, 746–759. https://doi.org/10.1111/j.1755-148X.2010.00757.x.

47. Boshuizen, J., Vredevoogd, D.W., Krijgsman, O., Ligtenberg, M.A., Blankenstein, S., de Bruijn, B., Frederick, D.T., Kenski, J.C.N., Parren, M., Brüggemann, M., et al. (2020). Reversal of pre-existing NGFR-driven tumor and immune therapy resistance. Nat. Commun. *11*, 3946. https://doi.org/10.1038/s41467-020-17739-8.

48. Boshuizen, J., and Peeper, D.S. (2020). Rational cancer treatment combinations: an urgent clinical need. Mol. Cell *78*, 1002–1018. https://doi.org/10.1016/j.molcel.2020.05.031.

49. Subramanian, A., Tamayo, P., Mootha, V.K., Mukherjee, S., Ebert, B.L., Gillette, M.A., Paulovich, A., Pomeroy, S.L., Golub, T.R., Lander, E.S., and Mesirov, J.P. (2005). Gene set enrichment analysis: a knowledge-based approach for interpreting genome-wide expression profiles. Proc. Natl. Acad. Sci. USA *102*, 15545–15550. https://doi.org/10.1073/pnas.0506580102.

50. Dobin, A., Davis, C.A., Schlesinger, F., Drenkow, J., Zaleski, C., Jha, S., Batut, P., Chaisson, M., and Gingeras, T.R. (2013). STAR: ultrafast universal RNA-seq aligner. Bioinformatics *29*, 15–21. https://doi.org/10.1093/bioinformatics/bts635.

51. Anders, S., Pyl, P.T., and Huber, W. (2015). HTSeq-A Python framework to work with high-throughput sequencing data. Bioinformatics *31*, 166–169. https://doi.org/10.1093/bioinformatics/btu638.

52. Kluin, R.J.C., Kemper, K., Kuilman, T., de Ruiter, J.R., Iyer, V., Forment, J.v., Cornelissen-Steijger, P., de Rink, I., ter Brugge, P., Song, J.Y., et al. (2018). XenofilteR: computational deconvolution of mouse and human reads in tumor xenograft sequence data. BMC Bioinf. *19*, 366. https://doi.org/10.1186/s12859-018-2353-5.

53. Young, L., Sung, J., Stacey, G., and Masters, J.R. (2010). Detection of Mycoplasma in cell cultures. Nat. Protoc. *5*, 929–934. https://doi.org/10.1038/nprot.2010.43.

54. Mootha, V.K., Lindgren, C.M., Eriksson, K.F., Subramanian, A., Sihag, S., Lehar, J., Puigserver, P., Carlsson, E., Ridderstråle, M., Laurila, E., et al. (2003). PGC-1α-responsive genes involved in oxidative phosphorylation are coordinately downregulated in human diabetes. Nat. Genet. *34*, 267–273. https://doi.org/10.1038/ng1180.

55. Hänzelmann, S., Castelo, R., and Guinney, J. (2013). GSVA: gene set variation analysis for microarray and RNA-Seq data. BMC Bioinf. *14*, 7.

CellPress
OPEN ACCESS

STAR★METHODS

KEY RESOURCES TABLE

REAGENT or RESOURCE	SOURCE	IDENTIFIER
**Antibodies**		
Phospho-STAT1	Cell Signaling Technology	Cat# 9167, RRID:AB_561284
IDO1	Cell Signaling Technology	Cat# 86630, RRID:AB_2636818
MITF	Abcam	Cat# ab12039, RRID:AB_298801
ATF4	Cell Signaling Technology	Cat#11815<br>RRID:AB 2616025
Vinculin	Sigma-Aldrich	Cat# V9131, RRID:AB_477629)
HSP90 apha/beta (H-114)	Santa Cruz Biotechnology	Cat# sc-7947
IFNγ blocking antibody (B27)	BioLegend	Cat#506532
CD3	eBioscience	16-0037-85; RRID: AB_468855
CD8	eBioscience	16-0289-85; RRID: AB_468927
**Bacterial and virus strains**		
E. coli strain: XL10-Gold Ultracompetent Cells	Internal stock	NA
1D3 virus	Internal stock	NA
**Chemicals, peptides, and recombinant proteins**		
Matrigel	Corning	356,230
Recombinant Human IFNy	Preprotech	300–02
Ficoll (1.078 g/mL)	Fisher Cientific	11,743,219
Retronectin	Takara	T100B
Epacadostat (*in vitro*)	SelleckChem	S7910
Epacadostat (*in vitro* and *in vivo*)	MedChem Express	HY-15689_4g
L-Tryptophan	Sigma Aldrich	T0254
IL-2	Slotervaart Hospital	Proleukin
InVivoMAb anti-mouse CD8a	BioXCell	BE0061
InVivoMAb rat IgG2b isotype control, anti-keyhole limpet hemocyanin	BioXCell	BE0090
IL-7	ImmunoTools	11,340,075
IL-15	ImmunoTools	11,340,155
Crystal Violet	Sigma	V5265
**Critical commercial assays**		
Tryptophan Assay Kit (Fluorometric)	Biovision	K557
Dynabeads™ CD8 Positive Isolation Kit	Themo Fisher	11333D
Bradford Protein Assay	Bio-Rad	5,000,006
Super-Signal West Dura Extended Duration Substrate	Themo Fisher	34,075
NEB® Golden Gate Assembly Kit (BsmBI-v2)	New England Biolads	E1602
**Deposited data**		
IFNγ-treated PDX cell lines RNA sequencing Data	This paper	GEO:GSE198460
TCGA (SKCM) RNA sequencing Data	TCGA	https://portal.gdc.cancer.gov/
Ribo-Seq data	This paper	Mendeley data: https://doi.org/10.17632/hd4h8fxdm9.1
Riaz anti-PD-1 treated cohort data	(Riaz et al., 2017)[35]	GEO:GSE91061
Raw imaging and quantification data	This paper; Mendeley data	Mendeley data: https://doi.org/10.17632/hd4h8fxdm9.1

(*Continued on next page*)

Continued

REAGENT or RESOURCE	SOURCE	IDENTIFIER
Gide anti-PD-1 and anti-PD-1+anti-CTLA4 treated cohort data	(Gide et al., 2019)[34]	ENA: PRJEB23709
Ji anti-CTLA-4 treatment cohort data	Ji et al., 2012[38]	Original article in supplementary table S3
Single cell sequencing data	Pozniak et al., 2022[36]	DOI: 10.110½022.08.11.502598.
Experimental models: Cell lines		
HEK293T	Internal stock	RRID: CVCL_0063
D10 (Endogenous HLA-A2, Endogenous MART-1)	Internal stock	
D10 (Exogenous HLA-A2, Exogenous MART-1)	Internal stock	NA
BLM (Exogenous HLA-A2, Exogenous MART-1)	Internal stock	RRID: CVCL_7035
A375 (Exogenous HLA-A2, Exogenous MART-1)	Internal stock	RRID:CVCL_0132
A375 (Endogenous HLA-A2, Exogenous MelanA)	Internal stock	RRID:CVCL_0132
888-Mel (Exogenous HLA-A2, Exogenous MART-1)	Internal stock	RRID:CVCL_4632
M026.X1.CL (Exogenous HLA-A2, Exogenous MART-1)	Internally generated	N/A
M026R.X1.CL (Exogenous HLA-A2, Exogenous MART-1)	Internally generated	N/A
M063.X1.CL (Exogenous HLA-A2, Exogenous MART-1)	Internally generated	N/A
SK-MEL-23 (Exogenous HLA-A2, Exogenous MART-1)	Internal stock	RRID:CVCL_6027
SK-MEL-28 (Exogenous HLA-A2, Exogenous MART-1)	Internal stock	RRID:CVCL_0526
Mel624 (Exogenous HLA-A2, Exogenous MART-1)	Internal stock	RRID:CVCL_8054
99.08 (Exogenous HLA-A2, Exogenous MART-1)	Internal stock	RRID:CVCL_VU43
SK-Mel-2 (Exogenous HLA-A2, Exogenous MART-1)	Internal stock	RRID:CVCL_0069
M048R.X1.CL (from fig2 not modified)	Internally generated	N/A
M026R.X1.CL (from fig2 not modified)	Internally generated	N/A
M027.X1.CL (from fig2 not modified)	Internally generated	N/A
M019R.X1.CL (from fig2 not modified)	Internally generated	N/A
M011-5R.X1.CL (from fig2 not modified)	Internally generated	N/A
M082.X1.CL (from fig2 not modified)	Internally generated	N/A
M080.X1.CL (from fig2 not modified)	Internally generated	N/A
M016.X1.CL (from fig2 not modified)	Internally generated	N/A
M061R.X1.CL (from fig2 not modified)	Internally generated	N/A
M063R.X1.CL (from fig2 not modified)	Internally generated	N/A
M029.X1.CL (from fig2 not modified)	Internally generated	N/A
M029R.X1.CL (from fig2 not modified)	Internally generated	N/A
M091.X1.CL (from fig2 not modified)	Internally generated	N/A
M026.X1.CL (from fig2 not modified)	Internally generated	N/A
M0002.X1.CL (from fig2 not modified)	Internally generated	N/A
A375-Melan A expression (*in vivo* experiment)	Internal Stock	RRID:CVCL_0132
B16F10 (Endogenous H2-Kb, Exogenous OVA)	Internal stock	RRID: CVCL_0159

(*Continued on next page*)

Continued		
REAGENT or RESOURCE	SOURCE	IDENTIFIER
Experimental models: Organisms/strains		
NSG mice	The Jackson Laboratory	Strain# 005557; RRID:IMSR_JAX:005,557
Black6 mice	Janvier	C57BL/6JRj
Recombinant DNA		
lentiCRISPR-v2	Addgene	RRID: Addgene_83480/RRID: Addgene_52961
psPAX	Addgene	RRID: Addgene_12260
pMD2.G	Addgene	RRID: Addgene_12259
lentiCas9-Blast	Addgene	RRID: Addgene_52962
Oligonucleotides		
sgIFNgR1 (Sequence = CGAACGACGGTACCTGAGGA)	Internal (Vredevoogd et al., 2019)[15]	NA
SgControl (Sequence = GGTTGCTGTGACGAACGGGG)	Internal (Vredevoogd et al., 2019)[15]	NA
Software and algorithms		
GraphPad Prism 9 (v9.0.0)	Graphpad Software Inc.	https://www.graphpad.com/scientific-software/prism/
R (v4.1.1)	R	https://cran.r-project.org/
RStudio (v1.4.1106)	RStudio, PBC	https://www.rstudio.com/
ImageJ	ImageJ	https://imagej.nih.gov/ij/
DESeq2 (version1.30.1)	Love et al., 2014.	https://bioconductor.org/packages/release/bioc/html/DESeq2.html#/
TCGAbiolinks (v2.22.3)	Colaprico et al., 2016	https://bioconductor.org/packages/release/bioc/html/TCGAbiolinks.html#/
javaGSEA version 2.2.3	Subramanian et al., 2005[49]	http://software.broadinstitute.org/gsea/index.jsp
STAR (v.2.6.0)	Dobin et al., 2013[50]	http://code.google.com/p/rna-star/.
HTSeq (v0.10.0).	Anders et al., 2015[51]	https://htseq.readthedocs.io/en/master/
GSVA in R (1.38.2)	Hänzelmann et al., 201,3[51]	https://bioconductor.org/packages/GSVA/
XenofilteR	Kluin et al., 2018[52]	https://github.com/PeeperLab/XenofilteR
Itreecount	NA	https://github.com/NKI-GCF/itreecount
Diricore	NA	https://doi.org/10.1038/nature16982

RESOURCE AVAILABILITY

Lead contact

Further information and requests for resources and reagents should be directed to and will be fulfilled by the lead contact Daniel S. Peeper (d.peeper@nki.nl).

Materials availability

The materials generated in this study did not generate new unique reagents.

Data and code availability

- RNA-Sequencing data from IFNγ-treated patient-derived cell lines have been deposited at GEO and are publicly available as of the date of publication. Accession numbers are listed in the key resources table.
- Original western blot images (and other source data related to this work) have been deposited at Mendeley and are publicly available as of the date of publication. The DOI is listed in the key resources table.
- This paper also analyzes existing, publicly available data. These accession numbers for the datasets are listed in the key resources table.
- All the R scripts used in this study are available upon request and without restriction to the lead contact.
- Any additional information required to reanalyze the data reported in this work paper is available from the lead contact upon request.

EXPERIMENTAL MODEL AND SUBJECT DETAILS

Human primary $CD8^+$ T cells

Buffy coats were purchased from Sanquin. Human PBMCs (peripheral blood mononuclear cells) were isolated by density gradient centrifugation using Ficoll (1.078 g/mL, Fisher Scientific #11743219). CD8 T lymphocytes were positively selected by CD8 Dynabeads (Thermo Fisher Scientific) following manufacturer's instructions. Anti-CD3 and anti-CD28 antibodies (eBioscience, 5 mg per well in 24-well plate format) were pre-coated for CD8 activation. After 48h of *in vitro* activation, T cells were transduced with retrovirus encoding 1D3 TCR. Transduction efficiency was assessed by FACS analysis and tumor cell-T cell ratios for co-culture were corrected for 1D3 TCR transduction efficiency. Multiple T cell donors (male and female origin) were used throughout this manuscript and can vary in killing efficiency between experiments.

Cell lines and culture conditions

All melanoma cell lines, including PDX-derived melanoma cell lines were obtained from the Peeper laboratory cell line stock. Cell lines derived from both male and female individuals were used. Cell lines were cultured in DMEM with 9% fetal bovine serum (FBS) (Sigma), 100U/mL penicillin and 0.1 mg/mL streptomycin (Gibco); HEK293T cells were used for virus production for ectopic expression of MITF, MelanA, GFP, HLA-A2, MART-1, sgRNAs or Ovalbumin. All cell lines were authenticated by STR profiling and regularly confirmed to be free of mycoplasma by PCR.[53] D10 and M0.26X1.CL cells have endogenous MART-1 and HLA-A2 expression; for Mel99.08, BLM and Mel624, cells we introduced MART-$1_{26\text{-}35}$ and HLA-A2 by viral transduction. MART-$1_{26\text{-}35}$ and HLA-A2 double-positive cells were sorted and seeded into 96-well plates at one cell per well. When single cells grew out, MART-1 and HLA-A2 expression were confirmed by FACS. For experiments in Figures 1A–1C, and 3A, all cell lines expressed MART-$1_{26\text{-}35}$ and HLA-A2 using lentiviral vectors and a hygromycin resistance cassette. After selection, the cells were used for follow up experiments. Cells were maintained in presence of hygromycin. D10 cells carrying either sgControl or a small guide RNA for IFNgR1 (sequences can be found on Resource table) were generated by lentiviral transduction and single cell clones were isolated for further experiments. For the *in vivo* experiment (Figure 1F and Extended data Figure 1D), A375 cells (which are endogenously HLA-A2-positive) ectopically expressing Melan-A were used. The *in vivo* experiment from Extended Data Figure 1E and 1F, B16-F10 melanoma cells expressing the protein ovalbumin (B16-OVA) were used.

Animal studies

Animal work procedures were approved by the animal experimental committee of the NKI and performed in accordance with ethical and procedural guidelines established by the NKI and Dutch legislation. 1 million tumor cells per mouse were mixed in 50uL PBS+50uL Matrigel (Corning) prior to injection. Vehicle and Epacadostat (MedChem) were administered after diluting in DMSO + Cremophor EL (2:1) with saline addition just before use (to a final concentration of 2:1:8). Adoptive cell transfer was performed in male NSG (JAX, bred at NKI) mice from 8–12 weeks. Briefly, human A375 melanoma cells ectopically expressing MelanA were injected subcutaneously into the flank. Five million transduced MART-1-specific T cells or control T cells ($CD8^+$ T cells that were not transduced with MART-1-specific TCR) were injected at d3 via tail vein followed by intraperitoneal injection of 100.000U of IL-2 (Proleukin) at d3-5 to support T cells. From d3 treatment with either vehicle or Epacadostat (100 mg/kg) was performed daily by oral gavage until tumors reached 1000 mm^3. In experiment shown in Figures 1F and S1D, mice were sacrificed when tumor size reached 1000 mm^3. For the experiments depicted in Extended Data Figure 1E, male C57BL/6JRj (8–12 weeks-old, from Janvier) mice were injected with B16-OVA cells (300.000 cells, also 1:1 PBS in Matrigel) and treated with epacadostat as described above, starting from day one. In Extended Data Figure 1F, each mouse received 100ug of CD8-depleting antibody or isotype control (BE0061, BE0090, from BioXCell), once a week prior to tumor cell injection until endpoint. $CD8^+$ T cell depletion was confirmed by flow cytometry. After that, the experiment was conducted as described for Extended Data Figure 1E. All animals are housed in disposable cages in the laboratory animal center of the NKI, minimizing the risk of cross-infection, improving ergonomics and obviating the need for a robotics infrastructure for cage-washing. The mice were kept under specific pathogen free (SPF) conditions. Tumor growth rates were analyzed by measuring tumor length (L) and width (W), and calculating volume through the use of the formula 1/2 × length (mm) × width (mm). The experiments were finished for individual mice either when the total tumor volume exceeded 1000 or 1400 mm3, when the tumor presented ulceration, in case of serious clinical illness, or when tumor growth assessment had been completed.

METHOD DETAILS

Lentivirus production

For virus production, HEK293T cells were transfected with the plasmid of interest and the helper plasmids (pMDLglpRRE, pHCMV-G and pRSVrev) with polyethylenimine. The day after, cells were refreshed and 24h later, culture supernatant was filtered and snap frozen for later infection. After overnight lentiviral infection, puromycin at 1 μg/mL (Sigma) or hygromycin at 5 μg/mL (Life technology) was added for selection.

Cytotoxicity assays

Cytotoxic assays were performed in 12-well plate format. Tumor cells (40–60k for IFNγ treatment and 150–200k for T cell co-cultures) were plated at day 0 and IFNγ (Preprotech) or T cells were added at day 1 in 1mL, and cells were stained with 0.1% crystal violet in

50% methanol after 4-6days after treatment or T cell co-culture (when the control reached 100% confluency) without media refreshment in the meantime. To investigate how tumor cells respond to tryptophan depletion, cell cultures were not replenished with fresh media (containing new tryptophan). IFNγ was diluted in water and kept at −80, and because of instability can vary in levels of phosphor-STAT1 induction throughout experiments. Viable tumor cells at the end of the assays were quantified by ImageJ or by crystal violet staining solubilization (10% acetic acid (Sigma). Absorbance was measured on an Infinite 200 Pro spectrophotometer (Tecan) at 595nm. Percentage of viable cells after treatment was calculated relative to the average of its own cell line control (untreated, set to 100%) and SD is shown based on each measured value in relation to the average control value. Epacadostat (2 μM, SelleckChem), exogenous tryptophan (Sigma) at 100ug/mL or IFNγ blocking antibody at 25ug/mL (Ultra-LEAF Purified anti-human IFNγ antibody from Biolegend) was always added together with either IFNγ or T cells. For protein and RNA analyses, cells were collected from 6 cm^2 or 10 cm^2 dishes and snap-frozen after harvesting for further analysis.

Immunoblotting and antibodies

Cells were lysed in RIPA (50mM TRIS pH 8.0, 150mM NaCl, 1% Nonidet P40, 0.5% sodium deoxycholate, 0.1% SDS) containing protease and phosphatase inhibitors (Halt - Thermofisher). Protein concentrations were measured by Bradford protein assay (Bio-Rad). Immunoblotting was performed on precast 4–12% bis-Tris gels (NuPage) and nitrocellulose membranes (GE Healthcare). Membranes were blocked in 4% milk powder and 0.2% Tween in PBS and incubated overnight with primary antibodies. Western blots were developed using Super-Signal West Dura Extended Duration Substrate (Thermo Fisher Scientific) and luminescence was captured by Chemidoc Imaging system (BioRad). Immunoblotting quantifications were done using ImageJ. The following antibodies were used: pSTAT1 (9167), IDO1 (86630), ATF4 (11815), from Cell Signaling; MITF (ab12039) from Abcam, Vinculin (V9131) from Sigma, HSP90 alpha/beta (H-114) from Santa Cruz.

RNA-sequencing and analysis

Low passage patient-derived melanoma cells were treated with 10 ng/mL of IFNγ for 24h, washed with cold PBS and and harvested for sequencing. Two independent biological replicates were performed. Total RNA was extracted using TRIzol reagent (15,596–018, Ambion life technologies) according to the manufactures protocol. The total RNA was further purified using the RNeasy Mini kit (74,106, Qiagen), including an on-column DNase digestion (79,254, Qiagen), according to the manufacturer's instructions. Quality and quantity of the total RNA was assessed by the 2100 Bioanalyzer using a Nano chip (Agilent, Santa Clara, CA). Total RNA samples having RIN>8 were subjected to library generation. Strand-specific libraries were generated using the TruSeq Stranded mRNA sample preparation kit (Illumina Inc., San Diego, RS-122-210½) according to the manufacturer's instructions (Illumina, Part # 15031047 Rev. E). The libraries were analyzed on a 2100 Bioanalyzer using a 7500 chip (Agilent, Santa Clara, CA), diluted and pooled equimolar into multiplex sequencing pools. The libraries were sequenced with 65 base single reads on a HiSeq2500 using V4 chemistry (Illumina Inc., San Diego). To remove the sequenced reads of mouse origin from the low passage patient-derived xenografted cell lines, sequenced reads were aligned in parallel using tophat-2.0.12 to mouse GRCm38 and human GRCh38. Subsequent alignments to reference were compared according to the edit distance by XenofilteR[52] to keep only sequences more likely human. For all the samples, the range of filtered reads is between 0.41 and 2.34%. Of the filtered human reads, per gene reads were counted using itreecount and annotated using Ensembl gtf GRCh38.82. Raw counts were pre-processed and normalized using DESeq2 (1.30.1). Gene sets were obtained from Molecular Signatures Database (v7.4) at GSEA. The gene set enrichment score (ES) for IFNγ hallmark and ATF4 reactome gene sets were obtained with GSEA on the signal to noise ratio between sensitive and resistant PDX cell lines, using the java application GSEA[54,49]

Ribosome profiling and analysis

D10 and Mel888 cell lines were treated with 5 ng/mL of IFNγ in the presence or absence of epacadostat (2uM) for 20h. Medium was harvested for tryptophan measurements showed in Figure 2E and cells were harvested on ice. The ribosome-protected fragment (RPF) libraries were constructed using SENSE Total RNA-Seq Library Prep Kit for Illumina (LEXOGEN) as previously described.[20] Sequencing was performed on a HiSeq 2000 System (Illumina). Diricore analysis of RPF, codon occupancy frequency between two conditions as indicated (e.g., IFNγ versus control) are compared with methodology described previously.[20]

Tryptophan measurement

Tryptophan concentration was measured from the supernatant of cultured melanoma cells after treatment with IFNγ or T cells in the presence or absence of epacadostat or exogenous tryptophan as indicated in the Figure legends. After supernatant collection, samples were boiled for 10 min at 100°C and kept at 4°C until measurement. A tryptophan Assay Kit by Biovision (K557) was used according to protocol and fluorescence was measured at Ex/Em = 370/440 nm on a Perkin Elmer EnVision plate reader.

Bioinformatic analysis

Raw read count data and clinical information for an anti-PD-1 treatment patient cohort[35] was downloaded from NCBI's GEO (GSE91061). Read count data was pre-processed and normalized using DESeq2 (1.30.0). Centering of the normalized gene expression data was performed by subtracting the row means and scaling by dividing the columns by the SD to generate a *Z* score. From the cohort, only the samples were selected for which both the samples before start of treatment were available as well as the matching

sample during treatment (n = 42). The change in average expression of the MITF-target genes (http://www.jurmo.ch/work_mitf.php) was calculated between pre-treatment and on-treatment samples. IFNγ signature was used from Ayers et al.[37]

In the anti-PD-1 and the combo treatment (anti-PD-1 & anti-CTLA-4) patient cohort, the RNA sequencing data was downloaded from the European Nucleotide Archive (ENA) under PRJEB23709.[34] The FASTQ files were aligned using STAR (v.2.6.0) with default setting on two-pass mode. The raw counts were generated using HTSeq (v0.10.0). Centering of the normalized gene expression data is performed by subtracting the row means and scaling by dividing the columns by the SD to generate a *Z* score. Only patient samples were selected if they were present in the pre- and on-treatment sets. IFNγ signature was used from Ayers et al.[37] In an anti-CTLA-4 treated cohort, genes showing decreased mean post-treatment expression in patients in the responder group[38] (data used for the analysis can be found in the original article in supplementary table S3) were used. MITF target genes (http://www.jurmo.ch/work_mitf.php) were selected, and expression values were scaled and plotted in a heatmap. Analysis showed in Figure 4A was done using the TCGA melanoma database. The enrichment of the gene sets (obtained using GSEA-msigdb) for individual samples was performed using single sample gene set enrichment analysis (ssGSEA) with GSVA in R (1.38.2).[55] In the anti-PD-1 treatment patient cohort, the ssGSEA of all responders (RECIST format: PR, CR) was compared to the ssGSEA of all non-responders (RECIST format: PD, SD). In the PDX dataset, the ssGSEA of the samples without treatment versus samples treated with IFNγ were compared. Single cell sequencing data of immuno-checkpoint treated melanoma cohort was provided and analyzed by Marine lab.[36] Other relevant information is available on original cited paper. Used codes with according references are Diricore,[20] DESeq2[51] (1.30.1/1.30.0), GSEA,[54,49] STAR[50](v.2.6.0), XenofilteR,[52] (HTSeq (v0.10.0)[51], GSVA in R (1.38.2),[55] Itreecount (https://github.com/NKI-GCF/itreecount). All the R scripts used in this study are available when request to the lead contact Daniel S. Peeper (d.peeper@nki.nl).

QUANTIFICATION AND STATISTICAL ANALYSIS

Statistical testing

In vivo tumor growth data was analyzed at the last time point before any mouse reached the endpoint, by one-way ANOVA with Šidák's Post-Hoc. For comparisons between two treatment groups in B16-OVA experiment, Mann-Whitney test was performed. For *in vitro* experiments, Mann–Whitney test or unpaired t-test was used for two conditions, depending on whether or not the data were normally distributed. Kruskal–Wallis or one-way ANOVA test were applied when two or more conditions were compared, depending on whether or not a normal distribution was observed. For ANOVA's multiple comparisons *Post-hoc* tests, Tukey's test was performed when comparing multiple groups against each other, or Dunnet's when comparing with the control condition. For Kruskal–Wallis post-hoc, Dunn's test was performed to correct for multiple comparisons. Correlations were calculated with Spearman coefficient. Calculations were always two-tailed. The analyses were performed with Prism Graphpad software v9 or R.

ADDITIONAL RESOURCES

Illustration

Parts of the graphical abstract figures were obtained from Servier Medical Art. Servier Medical Art by Servier is licensed under a Creative Commons Attribution 3.0 Unported License (creativecommons.org/licenses/by/3.0/).

Cell Reports Medicine, Volume *4*

Supplemental information

An adverse tumor-protective effect of IDO1 inhibition

Juliana C.N. Kenski, Xinyao Huang, David W. Vredevoogd, Beaunelle de Bruijn, Joleen J.H. Traets, Sofía Ibáñez-Molero, Sebastiaan M. Schieven, Alex van Vliet, Oscar Krijgsman, Thomas Kuilman, Joanna Pozniak, Fabricio Loayza-Puch, Alexandra M. Terry, Judith Müller, Meike E.W. Logtenberg, Marjolein de Bruijn, Pierre Levy, Pierre-René Körner, Colin R. Goding, Ton N. Schumacher, Jean-Christophe Marine, Reuven Agami, and Daniel S. Peeper

Supplemental file.

Figure S1

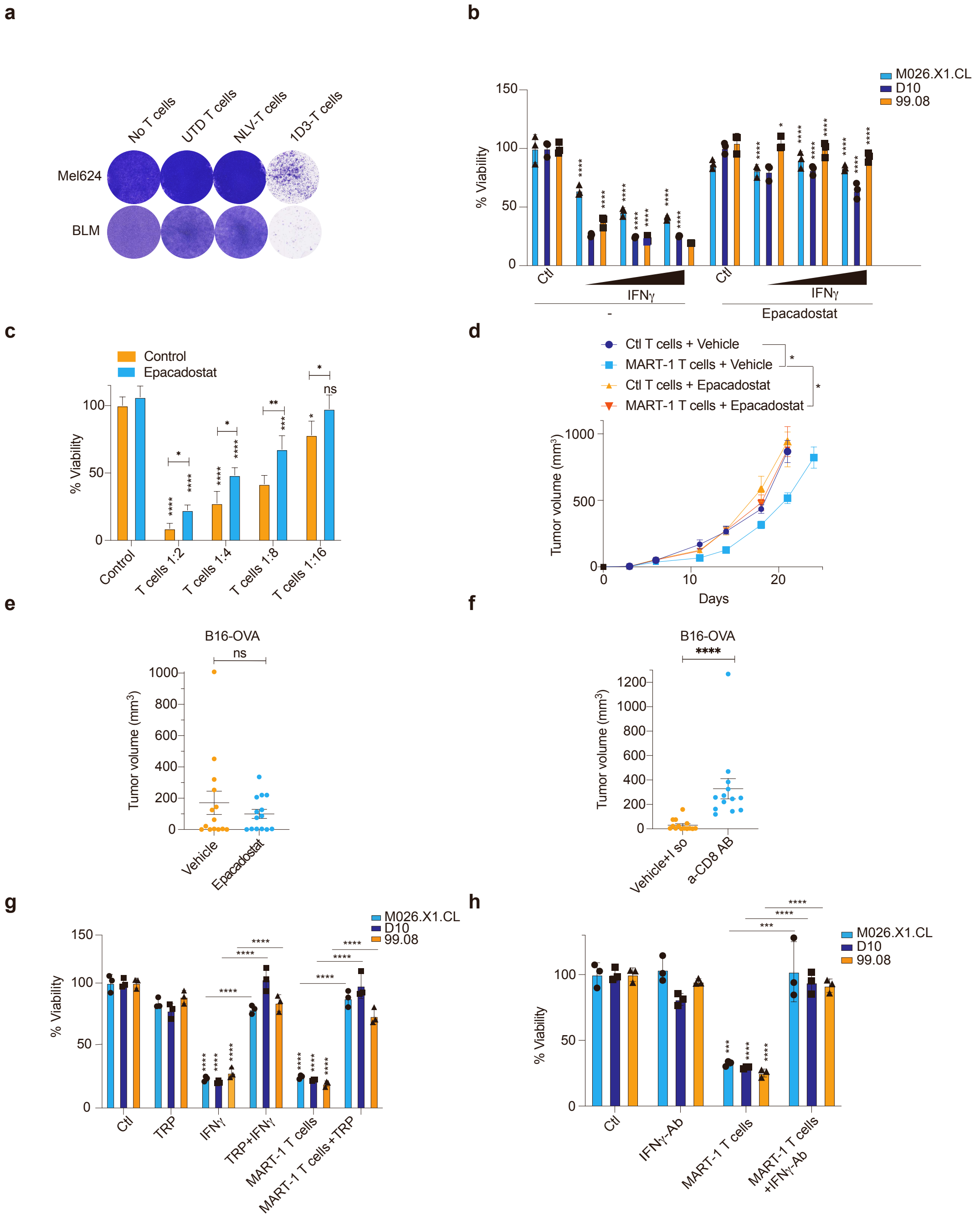

Figure S1 (related to Fig. 1). Tryptophan restoration by IDO1 inhibition protects tumor cells to T cell-mediated killing. a, Mel624 and BLM melanoma cell lines were exposed to 1D3-specific (MART-1) T cells for 24 hrs. Cells were stained with crystal violet after 6 days. Untransduced T cells and T cells containing the CMV-specific TCR (NLV) were used as controls to determine TCR-specific killing. **b**, Quantification of Fig. 1e. Statistical testing of the IFNγ only group was performed against its control, whereas in the epacadostat-treated groups it was compared to the corresponding IFNγ dose**. c,** A375-MelanA cells were co-cultured with MART-1 T cells at indicated effector to target ratios in the presence or absence of epacadostat (2uM). Cells were fixed and stained with crystal violet after 6 days. Graph shows quantification of the remaining viable melanoma cells. Statistical testing was done to compare both the effect of T cells against control (untreated) and T cells versus T cell+Epacadostat in each respective ratio. **d,** Growth curves of in vivo experiment shown in Fig. **1f** . **e,** B16-F10 melanoma cells expressing the model antigen ovalbumin (OVA) were injected into immunocompetent Black6 mice (n=14) and treated daily with either epacadostat (100mg/kg) or vehicle control by oral gavage. Tumors were measured three times a week and individual tumor sizes for the last time point when all the mice were alive. Statistical significance tested by Mann-Whitney. **f,** CD8-depleting antibody and isotype control were injected into immunocompetent Black6 mice until $CD8^{+}$ T cells were depleted (confirmed in the blood of treated mice by flow cytometry). B16-F10 melanoma cells expressing the model antigen ovalbumin (OVA) were injected after depletion was confirmed and tumors were measured three times a week and average tumor size +/- SEM are plotted in the graph. Graph shows individual tumor sizes for the last time point when all the mice from both groups were alive. Statistical significance tested by Mann-Whitney. In e, f, the experiment was done in parallel, but they have different time points due to distinct endpoints of each experiment. **g**, Quantification of Fig. 1h. Statistical testing of IFNγ and MART-1 T cell only groups was performed against their own controls. **h**, Quantification of Fig. 1j. Bars represent +/- SD for *in vitro* and SEM for *in vivo*. Statistical testing was performed on the three technical replicates by one-way Anova with Tukey's *Post-hoc* test. *= $p \leq 0.05$; **=$p \leq 0.01$; ***=$p \leq 0.001$; ****=$p \leq 0.0001$. One-way Anova with Šidák's Post-Hoc for **c, d**. *In vitro* experiments (except 1a) were performed in two biological replicates with three technical replicates each.

Figure S2

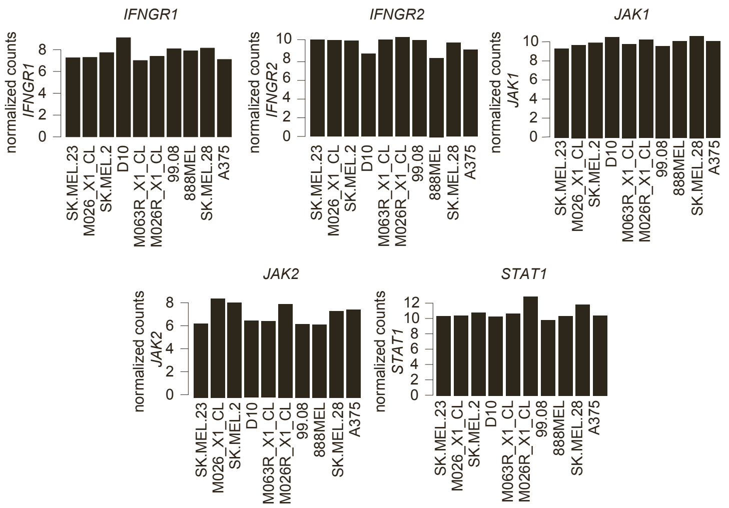

Figure S2 (related to Fig. 3). Expression of IFNγ pathway components by RNA-Sequencing. *IFNγR1, IFNγR2, JAK1, JAK2* and *STAT1* RNA levels were determined by RNA sequencing, which was performed in 2 independent biological replicates.

Figure S3

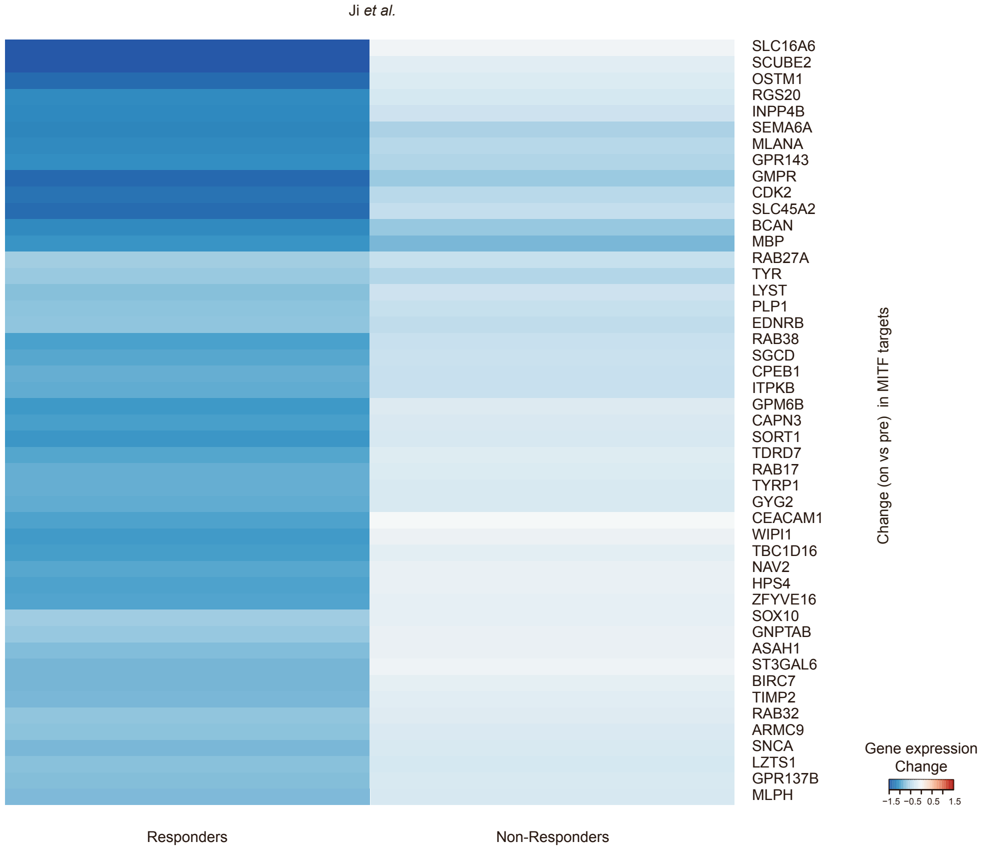

Figure S3 (related to Fig. 4). On treatment MITF downregulation predicts clinical outcome of anti-CTLA4-treated patients. Average change in gene expression upon treatment (pre- versus on-treatment) of MITF target genes in responders and non-responders to anti-CTLA-4 [S1].

References

[S1] Ji, R.R., Chasalow, S.D., Wang, L., Hamid, O., Schmidt, H., Cogswell, J., Alaparthy, S., Berman, D., Jure-Kunkel, M., Siemers, N.O., et al. (2012). An immune-active tumor microenvironment favors clinical response to ipilimumab. Cancer Immunology, Immunotherapy *61*, 1019–1031. 10.1007/s00262-011-1172-6.
